# High-content morphological profiling by Cell Painting in 3D spheroids

**DOI:** 10.1038/s41467-026-78596-5

**Published:** 2026-10-21

**Authors:** Christa Ringers, David Holmberg, Anders Larsson, Bomi Jung, Malin Jarvius, Anna Luchniak, Dan Rosén, Martin Johansson, Polina Georgiev, Brinton Seashore-Ludlow, Torkild Visnes, Åsmund Flobak, Jordi Carreras-Puigvert, Ola Spjuth

**Affiliations:** 1 Department of Pharmaceutical Biosciences, Uppsala University, Uppsala, Sweden; 2 Department of Clinical and Molecular Medicine, Norwegian University of Science and Technology (NTNU), Trondheim, Norway; 3 Department of Biotechnology and Nanomedicine, SINTEF Industry, Trondheim, Norway; 4 Chemical Biology Consortium Sweden, Science for Life Laboratory (SciLifeLab), Uppsala University, Uppsala, Sweden; 5 Discovery Sciences, BioPharmaceuticals R&D, AstraZeneca, Gothenburg, Sweden; 6 Department of Oncology-Pathology, Karolinska Institutet, Stockholm, Sweden; 7 Science for Life Laboratory (SciLifeLab), Karolinska Institutet, Stockholm, Sweden; 8 The Cancer Clinic, St Olav’s University Hospital, Trondheim, Norway; 9 Science for Life Laboratory (SciLifeLab), Uppsala University, Uppsala, Sweden; 10 PixlBio AB, Uppsala, Sweden

**Keywords:** High-throughput screening, High-throughput screening, Fluorescence imaging, Cellular imaging

## Abstract

Cell Painting is a popular high-content imaging assay that captures the shape and internal organization of 2D cell cultures at scale. While 3D cell cultures are more physiologically relevant models – especially for studying drug perturbations – they remain challenging to image because light scatters before it reaches the cultures’ interior. Here, we introduce a scalable method to adapt Cell Painting for 3D spheroids. We show that tissue-clearing, a technique that makes spheroids transparent, combined with a focusing routine that locates spheroids on the fly, enables Cell Painting at single-cell level inside intact spheroids. By profiling 52 clinically relevant drugs, we show that single-cell fingerprints clustered by biological pathway and resolved drug effects more robustly than those of flattened spheroid-wide views. Notably, the effect of two drugs, 5-fluorouracil and olaparib, nearly indistinguishable in 2D monolayers, diverged in spheroids. This finding was then corroborated by DNA-damage, cell-cycle, and gene-expression assays. The workflow runs on a spinning-disk confocal microscope in a standard 384-well plate format, making single-cell 3D profiling accessible to laboratories already performing Cell Painting.

## Abstract (web summary)

Cell Painting captures the internal organization of cells, but until now has been mostly confined to flat monolayers. Here, authors extend Cell Painting to single cells within 3D spheroids, demonstrating that drug responses which are alike in 2D cultures diverge within 3D cultures.

## Introduction

Phenotypic profiling and cellular assays play important roles in early drug discovery, offering valuable insights that have helped uncover new disease mechanisms^1,2^. One approach is morphological profiling, where a large number of quantitative metrics (features) – such as cell size, shape, and texture – can be extracted from individual cells within images, creating a unique fingerprint for every given condition^3,4^. This shift towards large-scale morphological profiling or phenomics allows for the exploration of less-characterized phenotypes, thereby facilitating the discovery of novel disease biomarkers^5–7^.

Cell Painting is a widely adopted standardized assay for morphological profiling of 2D monolayer cell cultures. By staining distinct cellular compartments and imaging in multiple channels, it enables the extraction of vast amounts of phenotypic information. This unbiased approach is powerful for predicting mechanisms of action^8,9^, identifying off-target effects^10^, and detecting phenotypes of environmental toxicants^11,12^. Moreover, a growing variety of open-source software^13,14^ and publicly available datasets^15^ enhances the ease of conducting and comparing Cell Painting experiments. Currently, the Cell Painting assay is primarily suited for 2D monolayers even though certain cellular functions – especially those that occur in three dimensions – remain challenging to capture in standard models^16–18^.

Three-dimensional (3D) cell cultures exhibit distinct biological characteristics compared to their 2D counterparts. In 3D, cells interact with neighboring cells and, depending on the specific model, the extracellular matrix (ECM)^19–22^. The rigid 2D surface of a flask, on the other hand, directs cells to form large focal adhesions instead of engaging in 3D interactions^21,23,24^. This often leads to the alignment of cytoskeletal actin filaments into stress fibers^25^ – a phenotype not typically observed in 3D cultures^21^. These differences in culturing methods do not only influence cell morphology but could also drive changes in cellular signaling^18^, such as promoting cell proliferation and impairing differentiation in 2D^24,26^, while reducing proliferation^27–29^ or diffusion of oxygen, nutrients, and waste in 3D^27,30^. In addition, interactions with the 3D environment – shaping both a cell’s morphology and internal signaling – could alter their drug response. The cell’s metabolic state also differs in 3D, potentially shifting their drug vulnerability^31,32^. Together, these make morphological profiling in 3D models increasingly relevant.

While considerable advances have been made towards scalable profiling in 3D^33–38^, current methods do not capture single-cell level phenotypic variations. Individual images are usually condensed into z-projections, effectively removing single-cell or lumen information, from which features are then extracted for further analysis^39,40^. Light scattering in samples more than a few cell layers thick poses a major hurdle to single-cell 3D imaging as it complicates downstream cell segmentation and feature analysis^41,42^. Recent advances using spinning disk confocal microscopy combined with either tissue-clearing or high Numerical Aperture (NA) water-immersion objectives show that high-throughput detailed 3D imaging is possible^43,44^. Nevertheless, its application in automated and multiplexed quantitative morphological profiling assays such as Cell Painting remains uncharted.

Here, we introduce a scalable method for Cell Painting in 3D spheroids. We show that in combination with tissue-clearing, the protocol can be effectively applied to 3D spheroids at the screening scale. Single-cell profiles resolved drug-induced phenotypes more robustly than spheroid-wide projections and recovered biological pathway structure; notably, 5-fluorouracil and olaparib were near-indistinguishable in 2D yet diverged in 3D, corroborated by EdU, γH2AX, and bulk RNA-seq. Altogether, this work establishes a framework for single-cell image-based phenotypic exploration in 3D spheroids.

## Results

### A cell painting workflow for 3D spheroids

**Fig. 1:**
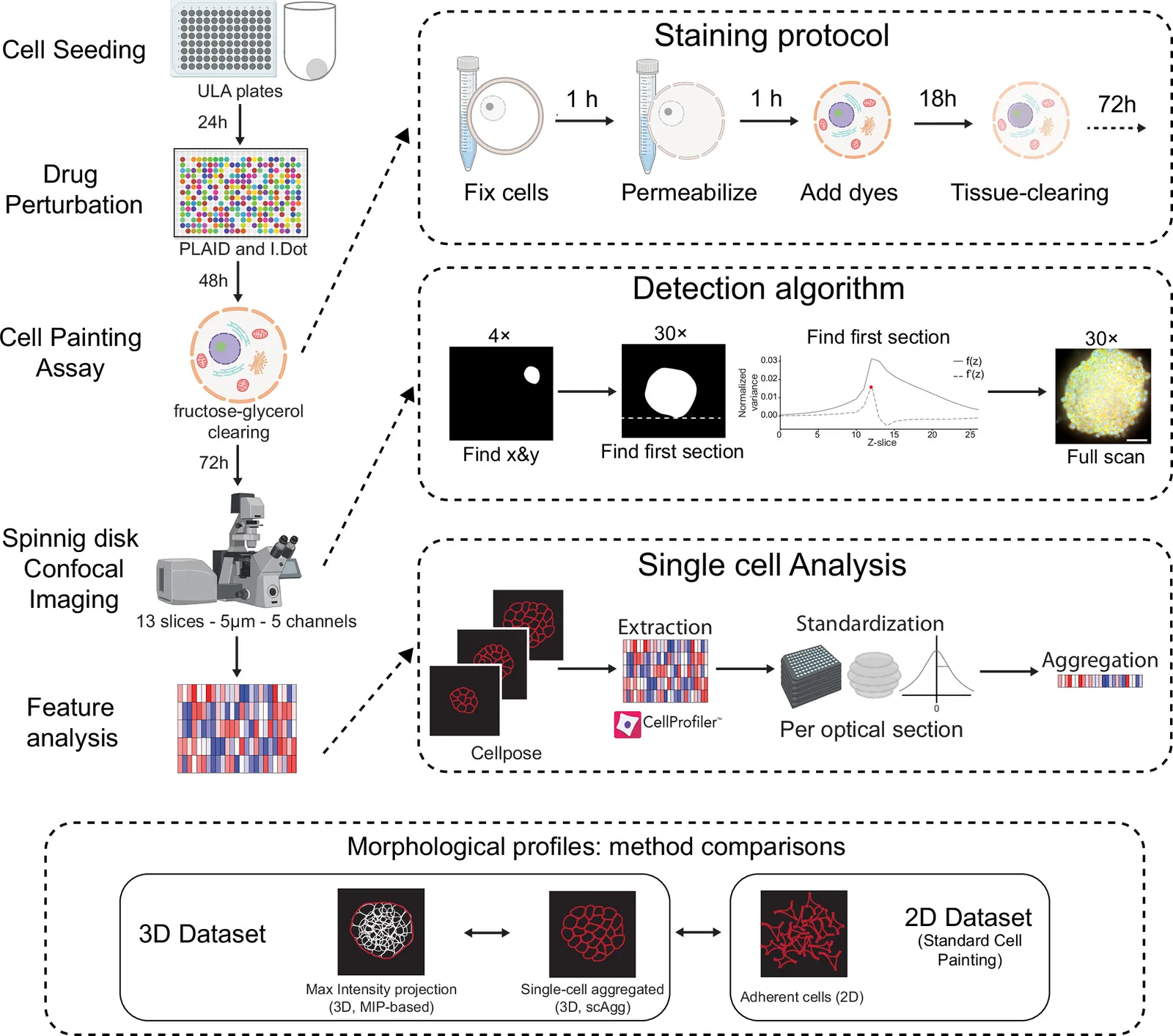
A workflow for Cell Painting of 3D spheroids. Schematic of the Cell Painting assay adapted to morphological profiling of spheroids (left). Cells are cultured in ultra-low attachment (ULA) plates (liquid-overlay) to form uniform spheroids. After 24 h, spheroids are exposed to a drug library in a plate layout designed with Plate Layouts using Artificial Intelligence Design (PLAID)^55^. Spheroids are then subjected to the Cell Painting assay, followed by spinning-disk confocal imaging and feature processing. Note the longer incubations and the fructose-glycerol clearing step. The staining protocol for spheroids in 3D: fixation of cells (1 h) is followed by permeabilization (1 h), dye incubation (18 h), and finally tissue-clearing by a fructose-glycerol mixture (72 h; top-right). The spheroid detection starts with a scan at 4× to find the X-and-Y position, followed by a Z-scan to find the first optical section of each spheroid, and finalized by multichannel high detail imaging of the spheroid. Each spheroid is imaged across twelve optical sections that are 5 µm apart (middle-right). The analysis pipeline consists of cell and nuclei detection by Cellpose, feature extraction by CellProfiler, per-section standardization and finally profile aggregation to collect per-well morphological profiles (bottom-right). Bottom panel: Downstream analysis compares the single-cell aggregated (scAgg) features to max intensity projection (MIP-based) features from the same spheroid dataset, as well as to features from a separately acquired 2D dataset. Schematic assembled by the authors in Adobe Illustrator. Created in BioRender. Ringers, C. (2026) https://BioRender.com/w85d529. One element adapted from NIAID Visual & Medical Arts, “HEK 293 Cells” (2024), NIAID NIH BioArt Source, bioart.niaid.nih.gov/bioart/198 (public domain).

We produced uniform spheroids using ULA plates^38^, which simplified cell seeding and enabled automation to improve assay throughput and robustness. To further improve dye penetration, we extended the incubation time to 18 h (Fig. 1).

Tissue-clearing homogenizes the refractive index between cellular and molecular components and the surrounding medium^42^. Originally developed for high-resolution imaging of large ex vivo organs, it can also be applied to spheroids and organoids for sub-cellular visualization^45,46^. In this study, we used an aqueous clearing solution comprised of fructose and glycerol, which achieves a high refractive index and provides good imaging results for spheroids and organoids with minimal preparation^45–48^.

**Fig. 2:**
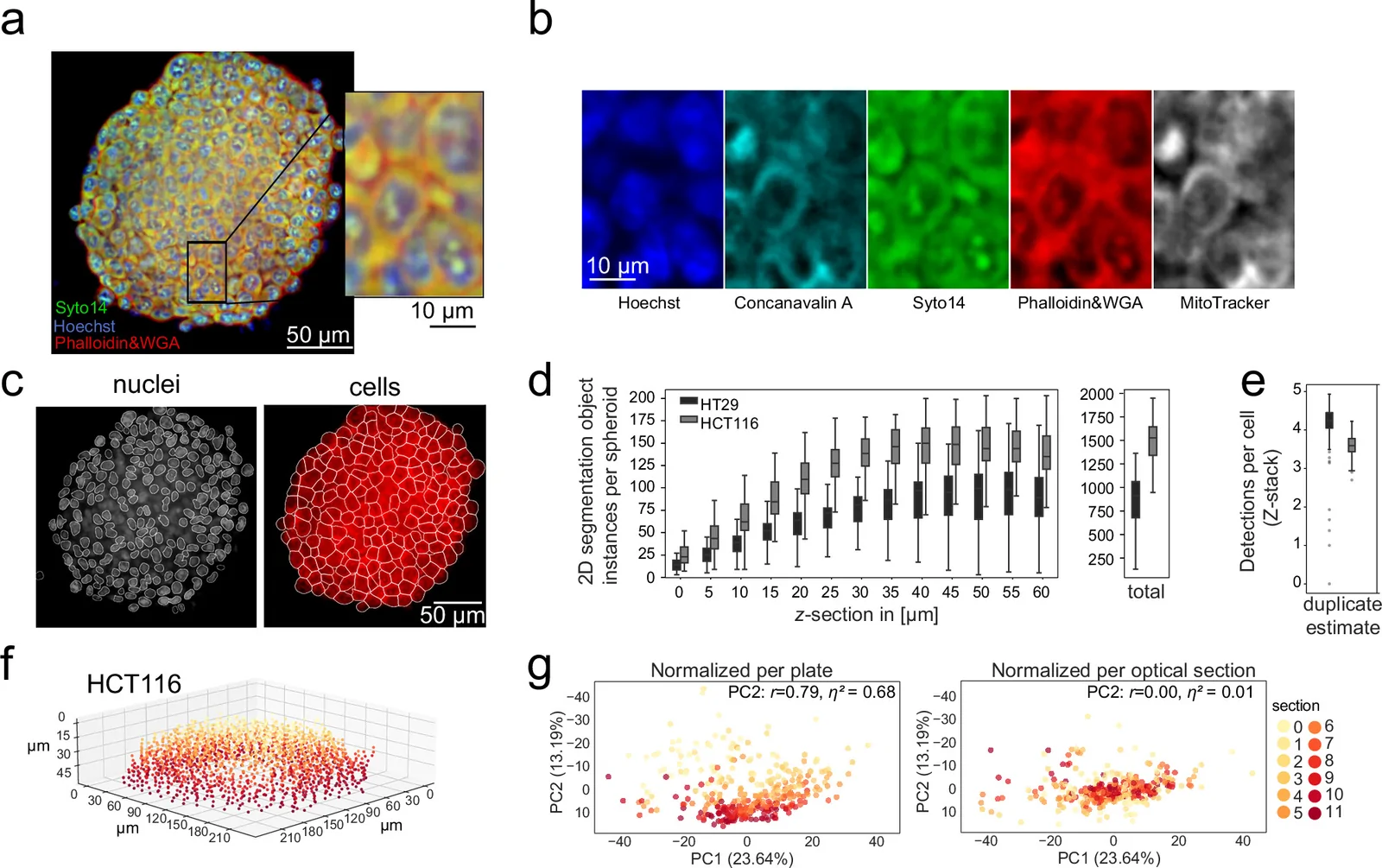
Towards reliable single-cell morphological profiles from cleared 3D spheroids. **a** Composite image of an HCT116 spheroid at 30 µm depth, showing nuclei (Hoechst, blue), RNA and cytoplasm (SYTO 14, green), and cytoskeleton, plasma membrane, and Golgi (phalloidin and wheat germ agglutinin, WGA; red). Scale bar is 50 µm, and spheroids were imaged at 30 × (0.227 µm/pixel). Inset: magnified view showing individual cells (scale bar, 10 µm). **b** Individual channels from the same acquisition: Hoechst (nuclei, blue), concanavalin A (endoplasmic reticulum, cyan), SYTO 14 (cytoplasm and RNA, green), phalloidin and WGA (cytoskeleton, plasma membrane, and Golgi, red), and MitoTracker (mitochondria, gray). **c** An example of nuclei (left) and cell segmentation (right) by Cellpose. Objects are outlined in white. Scale bar is 50 µm. **a**–**d** Number of 2D segmentation object instances per spheroid at each *z*-section for HCT116 (gray) and HT29 (black). Right: total instances summed across the full *z*-stack per spheroid. **e** Detections per cell across the *z*-stack (duplicate estimate), reflecting how many times the same cell is counted across optical sections. Each datapoint is one well at one section; *n* = 58 HCT116 and 56 HT29 DMSO wells. Box plots: center line, median; box bounds, 25th and 75th percentiles; whiskers, most extreme value within 1.5 × the interquartile range; outliers not shown. **f** Representative 3D distribution of detected cell positions in an HCT116 spheroid exposed to 0.1% DMSO. **g** Principal Component Analysis (PCA) of single-cell morphological profiles normalized per-plate (left) versus per-section (right). Both panels use identical PCA loadings (computed on the per-plate-normalized DMSO data; PC1 and PC2 explain 23.64% and 13.19% of total variance, respectively) to facilitate direct comparison. Section dependence was assessed by two-sided Pearson correlation of PC2 against optical section, with η² from a one-way ANOVA as a descriptive effect size (*n* = 348 well–section observations, 29 wells, no correction for multiple comparisons). Section-dependent effects are present before normalization (PC2: *r* = 0.79, η² = 68%, *p* = 1.1 × 10⁻^76^), but much less after (PC2: *r* = −0.00, η² = 1%, *p* = 0.96). Spheroid optical section (0–11) is color-coded in (**f**, **g**). Images in **a**–**c** are 16-bit, with display ranges of 190-1328 (Hoechst), 299-4332 (SYTO14), and 347-5876 (Phalloidin and WGA) out of 65535. Rolling-ball background subtraction (radius 50 pixels) was applied in an and **b** but not in **c**. Source data are provided as a Source Data file.

Optical sectioning with imaging techniques such as confocal or light-sheet microscopy has been widely used for visualizing complex 3D biological structures^36,43,49–51^. We took advantage of optical sectioning by spinning disk confocal to successfully image individual cells within cleared spheroids. The combination of long incubations, tissue-clearing, and optical sectioning allowed stains to diffuse deeper into the spheroid and be visualized during imaging. (Fig. 2a, b and Supplementary Fig. 1a, b).

Accurate and automated spheroid detection in 3D space is essential for consistent high-throughput imaging but remains a challenge due to well-to-well variability in spheroid positioning. To address this, we developed a custom on-the-fly detection algorithm that reliably determines each spheroid’s initial z position prior to imaging. Unlike methods relying on intensity, our algorithm leverages a sharp rise in image focus. Specifically, the algorithm monitors the FocusScore^14,52^ – a quantitative metric of image sharpness – and detects the sudden increase that occurs as a spheroid comes into view. The workflow begins with low-magnification X-Y positioning (4×), followed by high-magnification z-scanning (30 ×), and ends with multi-channel *z*-stack acquisition (Fig. 1). This process ensured consistent z position imaging across spheroids (Supplementary Fig. 1c), enabling acquisition of annotated *z*-sections for downstream analysis. To our knowledge, this is the first application of an open-source on-the-fly focus-based spheroid detection method tailored to high-content imaging.

For cell segmentation, we used Cellpose – a tunable deep learning-based model^53^ – to automatically segment cells from each acquired image section (Fig. 1). We reasoned that a 2D approach should still allow us to capture morphologies that are representative of their 3D biological structure. In this way, we could resolve individual cells inside spheroids (Fig. 2c), enabling 1425 ± 352 instances (2D segmentation objects) per spheroid in HCT116 and 854 ± 274 instances in HT29 (mean ± SD) (Fig. 2d–f and Supplementary Fig. 1d). A de-duplication estimate indicated a three- to fourfold duplication of each cell (Fig. 2e), which places the number of cells per spheroid at ~ 400 (HCT116) and ~ 330 (HT29). From the original segmentations, we could then extract > 2000 morphological features using CellProfiler.

Despite these optimizations, a depth-dependent decrease in intensity and contrast was still noticeable (Supplementary Fig. 1a, b). To address this, we normalized each optical section against the negative controls imaged at the same depth (Fig. 1). Depth-dependent effects present before normalization (Fig. 2g, left; *r* = 0.79, *η*² = 68%, *p* = 1.1 × 10⁻⁷⁶) were mitigated afterwards (Fig. 2g, right; *r *= − 0.00, η² = 1%, *p* = 0.96). Corrected morphological profiles were used for downstream analysis.

While some aspects of the workflow depend on specific imaging systems, such as the spinning-disk, type of objective and its lasers, the underlying principles – tissue-clearing, automated segmentation, and normalization – can be adapted to other platforms. These advancements maintain the efficiency and scalability of the original Cell Painting assay while enabling 3D profiling in spheroids.

### Single-cell level profiling captures phenotypic changes with greater resolution compared to spheroid-wide profiling (MIP)

To evaluate our 3D Cell Painting workflow and compare morphological responses across culture formats, we generated two complementary datasets and analyzed them through three analytical pipelines. The 3D dataset comprised colorectal cancer spheroids (HCT116 and HT29) profiled with our 3D Cell Painting workflow, from which we extracted morphological profiles using either spheroid-wide maximum intensity projections (MIP-based) or single-cell aggregates (scAgg). An independently acquired 2D adherent dataset of the same cell lines and compounds, profiled with standard Cell Painting, served as the reference for culture-format comparisons (Fig. 1, bottom panel). Both datasets used 52 clinically relevant drugs and pre-clinical compounds, from now on referred to as drugs, with 4 replicates per condition and 20 DMSO wells per plate (Supplementary Fig. 2a and Supplementary Data 1). A summary of the assay, compound library, screen design, and post-screen analysis is provided in Supplementary Data 2. We also included the phenotypic reference compounds etoposide and fenbendazole^54^. In short, cells were seeded in ULA plates 24 h before exposure to allow for spheroid formation. We designed effective plate layouts using the constraint programming tool PLAID^55^. After 48 h of drug exposure, we performed our 3D Cell Painting workflow followed by fructose-glycerol clearing for 72 h, making the entire protocol span one week. We successfully performed automated staining and imaging of 12-plane spheroids across six 384-well plates in this single experimental run.

**Fig. 3:**
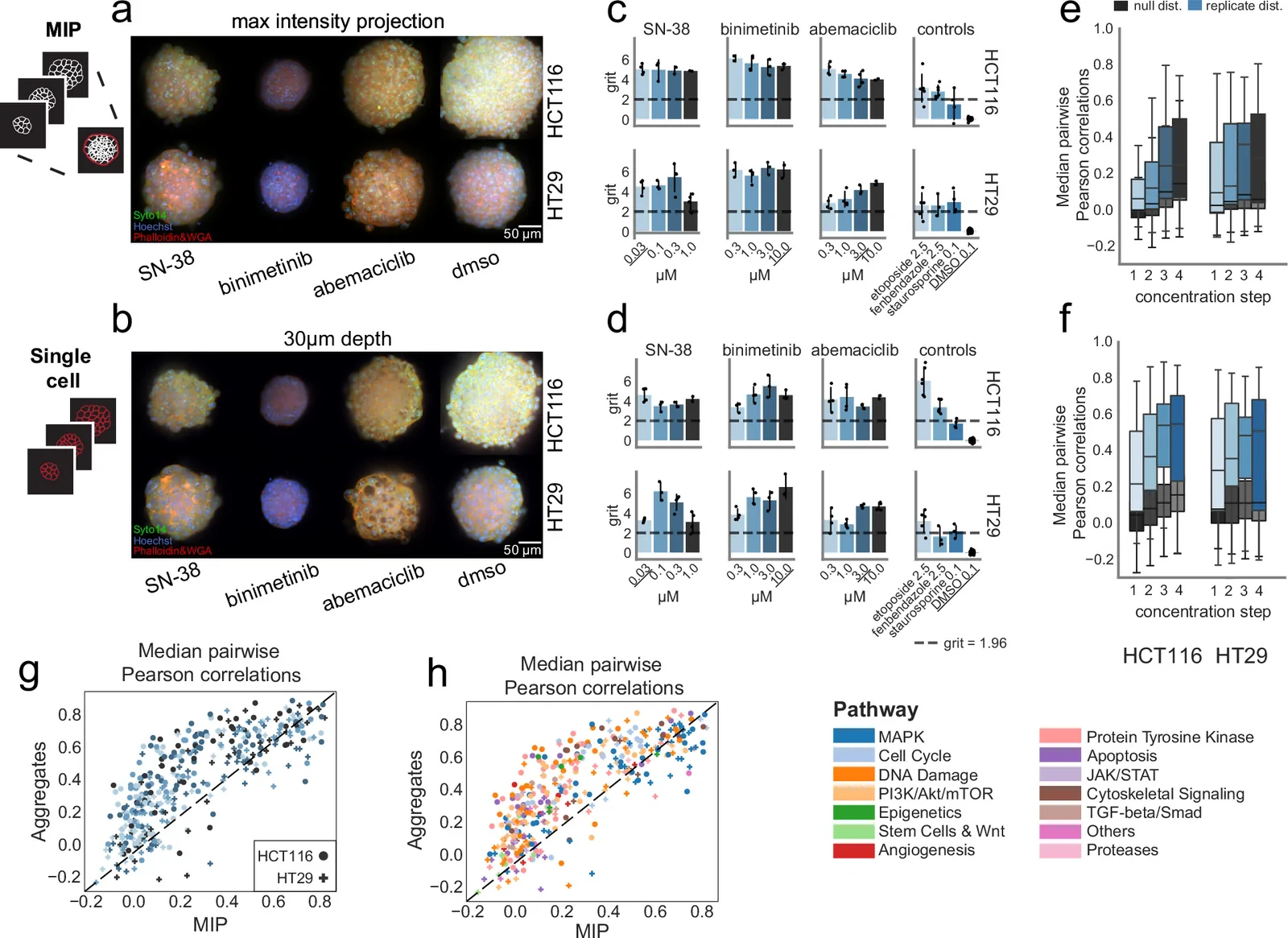
Comparing the quality of single-cell level profiles to spheroid-wide (MIP-based) morphological profiles. **a** Representative examples of HCT116 (top) and HT29 (bottom) max intensity projections (MIPs) for the compounds SN-38 (0.03 µM), binimetinib (10 µM), abemaciclib (3.0 µM), and DMSO (0.1%), as well as for (**b**) a single section at 30 µm depth. SYTO 14 (RNA) is shown in green, Hoechst (nuclei) is shown in blue, and phalloidin/WGA (cytoskeleton and golgi) are shown in red. Scale bars are 50 µm; images were acquired at 30 × (0.227 µm/pixel). Images are 16-bit, with display ranges of 190-1328 (Hoechst), 299-4332 (SYTO14), and 347-5876 (Phalloidin and WGA) out of 65535. No rolling-ball background subtraction was applied. **c**, **d** Grit scores measuring phenotypic effect are shown for MIP-based (**c**) and aggregated (**d**) profiles. Each bar is one compound–dose perturbation, 4 wells; error bars, mean ± 1 s.d. The dashed horizontal line marks the grit > 1.96 threshold. **e**, **f** Null and median pairwise replicate correlations distributions across dose steps for both HCT116 and HT29 cell lines. Null distributions are in grays and replicate in blues. Box plots (both series): center line, median; box bounds, 25th and 75th percentiles; whiskers, most extreme value within 1.5 × the interquartile range; outliers not shown. Each point is one compound–dose perturbation (4 wells); *n* = 41–52 per box, 208 per null. **c**–**h** pre-filter, treatment wells only. **g**, **h** Comparison of median pairwise replicate correlations of MIP-based and scAgg profiles in both HCT116 and HT29 cell lines. Data points are compound–dose perturbations (*n* = 383; 196 HCT116, 187 HT29), color-coded for (**g**) dose, or (**h**) pathway. Source data are provided as a Source Data file.

We then profiled those spheroids using Cellpose and CellProfiler (Fig. 1). Feature processing, however, differs between 2D and 3D. In 2D Cell Painting, single-cell data are commonly aggregated into median cell features^3,56^, whereas high-content experiments on 3D cultures often rely on z-projections for feature extraction^33–35,37–39^. One such technique is the maximum intensity projection (MIP), which selects the brightest pixel across a *z*-stack (Fig. 3a). Yet, MIPs can introduce artifacts, such as misrepresented object positioning, and lose relevant single-cell information^39,40^.

To address these limitations, we extracted morphological features using two approaches: MIP at the spheroid-wide level (Fig. 3a) and scAgg at the individual cell level (Fig. 3b). Several drug treatments induced visible phenotypic shifts. For example, spheroids exposed to binimetinib were smaller and appeared to have compact cytoplasmic regions; SN-38 treatment resulted in fewer but larger cells; and abemaciclib exposure induced structures reminiscent of lysosome-derived vacuoles^57^ (Fig. 3a, b and Supplementary Fig. 2b). The drug-induced changes also altered spheroid optical properties, introducing treatment-related variation that could bias per-section normalization. We therefore checked whether equivalent sections across wells were being compared and whether segmentation was affected. Focus peaked at similar depth (20–25 µm) across treatments and channels, despite differences in overall signal (Supplementary Fig. 2c). Segmentation quality, validated against expert annotations on compound-treated spheroids, decreased with depth and was lowest for binimetinib (Supplementary Fig. 2d). However, these errors propagated minimally to the extracted features (Pearson *r* > 0.8; Supplementary Fig. 2e). We then quantified the treatment-induced morphological shifts by calculating grit scores for both the MIPs and scAgg profiles, indicating how much more similar a drug is to its replicates than to the negative controls^58–60^. A grit of 1.96 corresponds to the two-sided 95% critical value, serving as a threshold for meaningful phenotypic change. In HCT116 cells, 38 drugs in MIP-based profiles (*µ *= 2.01 ± 1.78) and 37 in scAgg (*µ *= 1.86 ± 1.95) exceeded this threshold. For HT29 cells, 33 (MIP, *µ *= 1.92 ± 1.95) and 32 (scAgg, *µ *= 2.10 ± 2.26) drugs showed significant shifts (Fig. 3c, d). The results indicate that both preprocessing strategies are comparable in detecting morphological changes in perturbed spheroids.

To evaluate the resolution and technical-replicate reproducibility of the assay, we used the median pairwise Pearson correlation between replicate profiles relative to a null distribution generated by randomly sampling non-replicates^61,62^. We find that the replicate correlation increases with concentration (Fig. 3e, f), is higher for the HCT116 cell line, and slightly lower than previously published values – although values may fluctuate depending on compounds included in an experiment^61,62^. When comparing replicate correlation between MIP-based and scAgg profiles, we indeed observe higher values when extracting features from single cells (Fig. 3g, h). This effect does not seem to be carried by cell line, concentration (Fig. 3g), or pathway (Fig. 3h; annotations in Supplementary Data 1).

This indicates that while both MIP-based and scAgg profiles are different and distinguishable from negative controls, it is the scAgg profiles that can be discriminated from each other. In other words, those profiles have a better resolution.

### Single-cell morphological profiles facilitate the detection of biologically relevant clusters

**Fig. 4:**
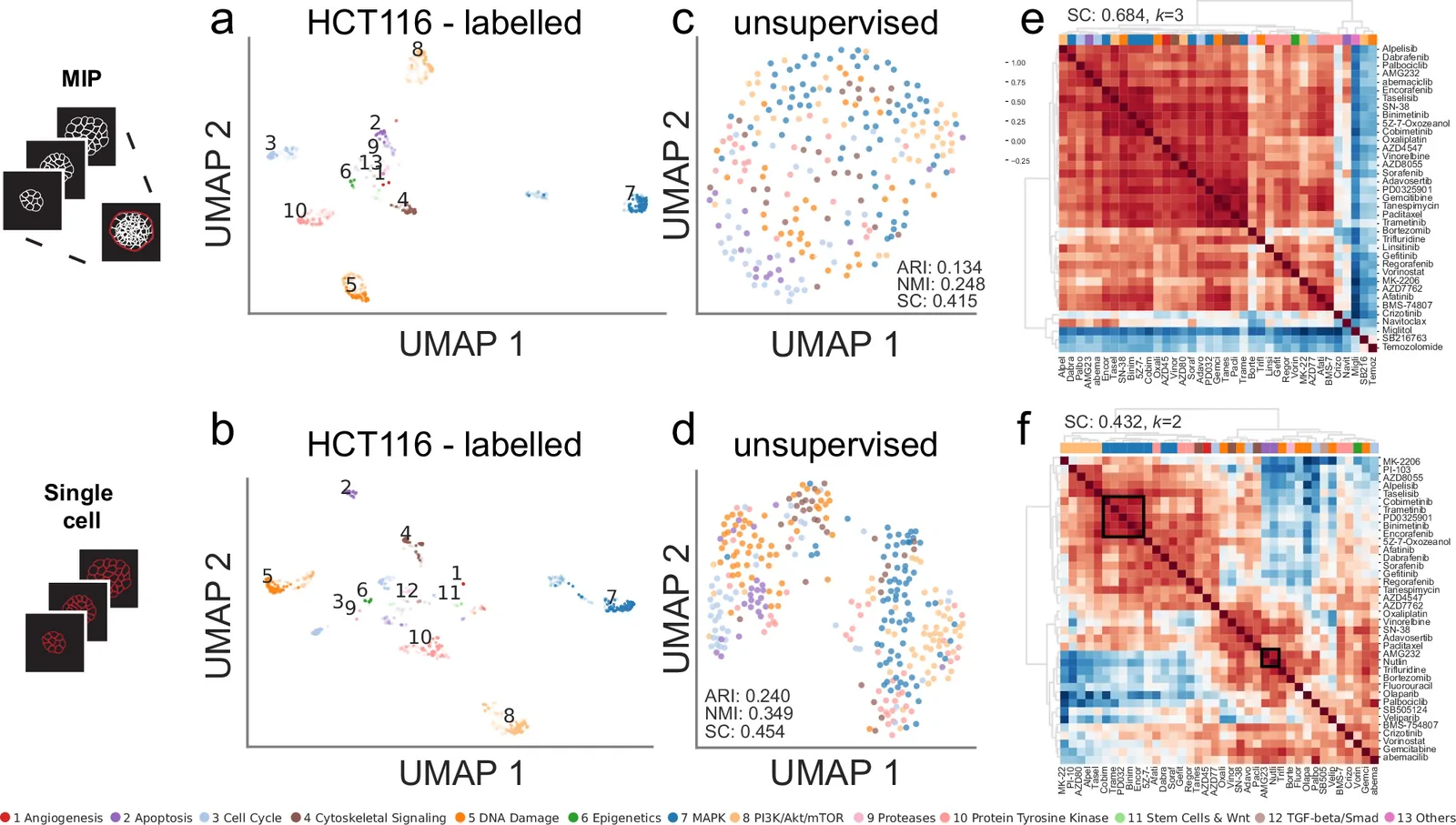
Single-cell morphological profiles facilitate the detection of biologically relevant clusters. **a** Semi-supervised UMAPs of MIP-based and (**b**) scAgg morphological profiles. **c** Unsupervised UMAPs of MIP-based and (**d**) scAgg morphological profiles, with clusters numbered 1–k in the plot. Pathway enrichment scores (Adjusted Rand Index; ARI, Normalized Mutual Information; NMI) and cluster cohesion (SC) are reported alongside. **e** Heatmaps displaying hierarchical clustering of pairwise cosine similarities for MIP-based and (**f**) scAgg profiles. Ward linkage and Euclidean distance was used to create the dendrogram; the optimal number of clusters (MIP: *k* = 3; scAgg: *k*=2) was selected by maximizing the average silhouette score (MIP: SC = 0.684, scAgg = 0.432). In all panels, pathways are color-coded using the same colormap. All analyses are based on HCT116 spheroid perturbations with a grit score > 1.96. Source data are provided as a Source Data file.

Morphological profiling via Cell Painting has been used to profile compounds based on their bioactivity, assess drug safety, and predict compound mechanism of action (MoA)^62–65^. A central aspect of these tasks is leveraging morphological profiles to identify phenotypically similar clusters of compounds. To evaluate the potential of our MIP-based and scAgg profiles and discriminate drug clusters from perturbed 3D spheroids, we first explored Uniform Manifold Approximation and Projection (UMAP)^66^ – an unsupervised method commonly used for downstream analysis of biological features^67^. We included class labels – here pathways – in the clustering algorithm to account for data variability. This allowed us to identify separate clusters per pathway in the UMAPs for MIP-based and scAgg profiles in HCT116 and HT29 cell lines (Fig. 4a, b and Supplementary Fig. 3a, b). To assess pathway-level enrichment in the data, we rendered unsupervised UMAPs (Fig. 4c, d and Supplementary Fig. 3c, d) and calculated clustering metrics. For HCT116 profiles, both MIP-based and scAgg embeddings showed modest but significant pathway enrichment (ARI: 0.134 vs. 0.240, NMI: 0.248 vs. 0.349; *p* < 0.001). HT29 profiles followed a similar pattern (ARI: 0.196 vs. 0.276, NMI: 0.313 vs 0.383; *p* < 0.001), indicating detectable pathway structure despite technical noise, multiple targets grouped within pathways, and an unbalanced pathway representation in this dataset.

To complement our UMAP analysis, we performed hierarchical clustering of pairwise cosine similarities, retaining only drugs with grit > 1.96 (Fig. 4e, f and Supplementary Fig. 3e, f). In HCT116, MIP-based clusters were more cohesive than scAgg profiles (SC = 0.684 and 0.432 at *k* = 2) but showed little in biological specificity (ARI = 0.006), whereas scAgg profiles better recovered pathway structure (ARI = 0.159). For scAgg profiles, one cluster primarily includes kinase inhibitors (MEK, PI3K, and PTK), and the other contains a more heterogeneous set including DNA-damaging agents. As a specific example, the two CDK4/6 inhibitors abemaciclib and palbociclib showed highly similar phenotypes (cosine sim = 0.89) in both cell lines. Together, these results indicate that scAgg profiles recover a biologically meaningful, mechanism-of-action-aligned structure.

### 3D Cell Painting is robust to protocol variation and transferable across sites

We next assessed how sensitive the workflow is to protocol choices. Using HCT116 spheroids, we varied axial sampling (*z*-step size and post-hoc subsampling), seeding density, clearing, and magnification, and compared each condition’s replicate correlations with a matched non-replicate null (Supplementary Fig. 4). Most comparisons included six compounds and could only detect large effects, whereas the *z*-section subsampling (*c* = 50) was well powered. Axial sampling was forgiving; replicate correlations exceeded the null for every z-section subset tested, including single planes, and subsets of 6 or 9 planes performed equivalently to the full 12-plane stack (Supplementary Fig. 4a). A finer acquisition using a 1.5 µm *z*-step did not improve reproducibility (Supplementary Fig. 4b); instead, it showed stronger intensity decay with depth in the longer-wavelength channels, consistent with photobleaching, as well as fewer detected cells (Supplementary Fig. 4g, h). Replicates of the largest spheroids (810 cells/well) failed to exceed the null (Supplementary Fig. 4c, f). Clearing did not affect reproducibility but increased cell detection at depth, with 20% glycerol (v/v)/40% fructose (w/v) performing as well as 40% glycerol (v/v)/40% fructose (w/v) (Supplementary Fig. 4d, f, h). Finally, 30× magnification performed comparably to 10 ×, whereas 40 × failed to exceed the null (Supplementary Fig. 4e, f).

To explore whether the workflow transfers to another site, we ran it at AstraZeneca, Gothenburg, on a Yokogawa microscope instead of the Nikon used at Uppsala University. There, we could also compare an air with a water immersion objective (Supplementary Fig. 4i). Spheroids were exposed to six reference compounds at ten concentrations, and the compounds clustered reliably (Supplementary Fig. 4j). Water immersion images appeared sharper, with a higher mean FocusScore at 30 µm depth (air: *µ* = 1639 ± 272, WI: *µ* = 2458 ± 793), but fewer spheroids were detected (46.43% versus 66.1%), and clustering quality was correspondingly lower (Supplementary Fig. 4k). The workflow therefore transferred across sites and microscopes and was robust to most protocol variations tested.

### Morphological profiles reveal different responses to DNA-damage-response drugs in spheroids versus adherent cells

**Fig. 5:**
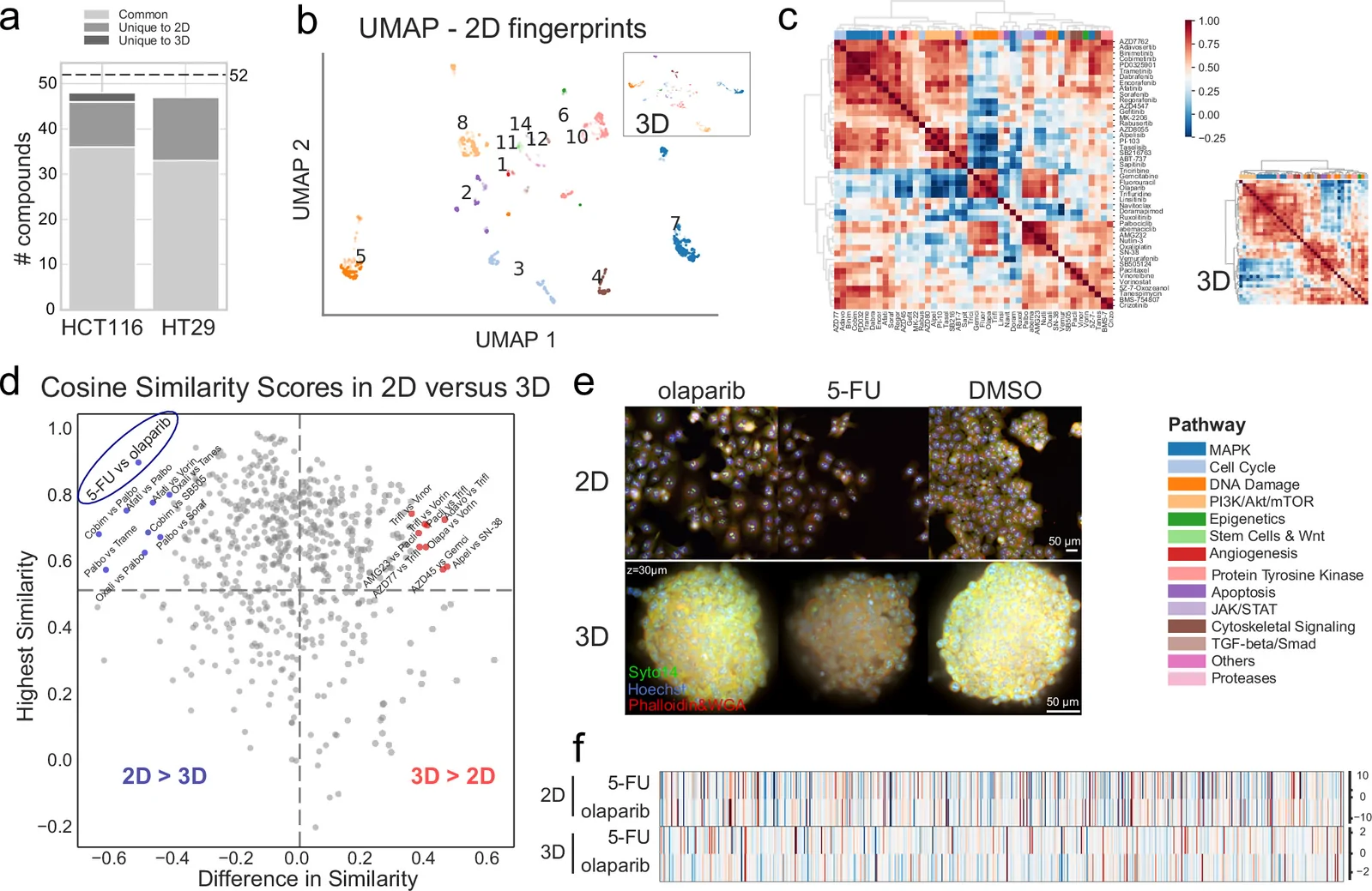
Morphological profiles reveal different responses to DNA-damage-response compounds in spheroids versus adherent cells. **a** A quantification of the number of drugs with grit > 1.96 for cell lines HCT116 and HT29. Color-coded are the drugs unique to each culture format, as well as the non-unique drugs. The total amount of drugs is 52. **b** Semi-supervised UMAPs of 2D morphological profiles. **c** Heatmap displaying hierarchical clusters of cosine similarities for 2D profiles. **d** Comparison of cosine scores between pairs of compounds in 2D and 3D. The x-axis shows the difference in similarity scores (3D – 2D), while the y-axis shows the highest similarity score across both modalities for each pair. Pairs whose absolute between-format difference (3D – 2D) fell in the top 5% (95th percentile) across all matched pairs were flagged as divergent; among these, pairs with a cosine score of at least 0.5 in either 2D or 3D were labeled. **e** Representative images of cells in 2D (top) and 3D (bottom) exposed to olaparib (3 µM), 5-fluorouracil (5-FU) (10 µM), and the negative control DMSO (0.1%). SYTO 14 is green, phalloidin/WGA is red, and Hoechst is blue. 2D images were acquired at 20 × (0.34 µm/pixel) and 3D images at 30 × (0.227 µm/pixel) from a 30 µm depth. Images are 16-bit, with display ranges of 190-1328 (Hoechst), 299-4332 (SYTO14), and 347-5876 (Phalloidin and WGA) out of 65535. No rolling-ball background subtraction was applied. **f** Preprocessed morphological profiles are shown for 2D (top) and 3D (bottom) exposed to 5-FU (10 µM) and olaparib (3 µM). Scale bars are 50 µm. Source data are provided as a Source Data file.

Having established morphological profiling in 3D spheroids, we next asked whether drug-induced phenotypes differ between culture formats. Prior work shows viability differences between 2D and 3D^68–70^, but morphological response patterns remain largely unexplored. We therefore treated adherent HCT116 and HT29 cells with the same 52-drug panel and profiled them using standard Cell Painting. To evaluate response strength, we computed the grit scores for both formats. In 2D, 46 drugs in HCT116 (*µ *= 3.90 ± 2.65) and 47 in HT29 (*µ*=3.91 ± 2.91) exceeded the grit threshold (Fig. 5a). In comparison to 3D (scAgg), this number was 37 (*µ *= 1.86 ± 1.95) and 32 (*µ *= 2.10 ± 2.26). Since the grit score reflects both effect size and measurement noise, the lower 3D grit scores could reflect technical challenges of volumetric imaging, weaker biological responses, or both.

We then examined whether drugs clustered similarly in both formats. In UMAP embeddings, drugs targeting MAPK and PI3K/Akt/mTOR pathways formed distinct clusters in both 2D and 3D. DNA-damaging agents, however, appeared clustered in 3D but split in 2D (Fig. 5b and Supplementary Fig. 5a). Hierarchical clustering uncovered a similar structure: a kinase inhibitor cluster (MAPK, PI3K/Akt/mTOR) in both formats and a DNA-damaging agents cluster that split only in 2D (Fig. 5c and Supplementary Fig. 5b). UMAP and hierarchical clustering are qualitative, however, and do not identify which drug relationships change between formats.

To quantify these differences, we computed pairwise cosine similarities between drug profiles separately for 2D and 3D. Comparing similarities rather than the profiles themselves avoids cross-dataset batch effects^71,72^. We then focused on the 5% of drug pairs whose similarity differed most between 2D and 3D (95th percentile of absolute similarity differences; Fig. 5d and Supplementary Fig. 5c), as these indicate format-specific responses. Several of these pairs included at least one DNA-damage drug, consistent with prior reports of differential responses to such agents in 2D and 3D^69,73–77^.

For instance, 5-fluorouracil (5-FU) and olaparib were morphologically similar in 2D but distinct in 3D (Fig. 5d), suggesting a format-dependent difference in their cellular response. The divergence was consistent across doses (Supplementary Fig. 5d) and was not unique to this pair (Supplementary Fig. 5e). It was also detectable only in single-cell profiles: neither drug exceeded the grit threshold in MIP-based profiles (highest dose: 5-FU = 1.05, olaparib = 0.98). Both drugs were previously reported to induce no measurable cytotoxicity in either format by metabolic activity assays^68^, yet both triggered clear phenotypic responses (Fig. 5e, f). Morphological profiling, therefore, captured drug effects beyond viability.

### Molecular profiling confirms different responses to DNA-damage-response agents in spheroids

**Fig. 6:**
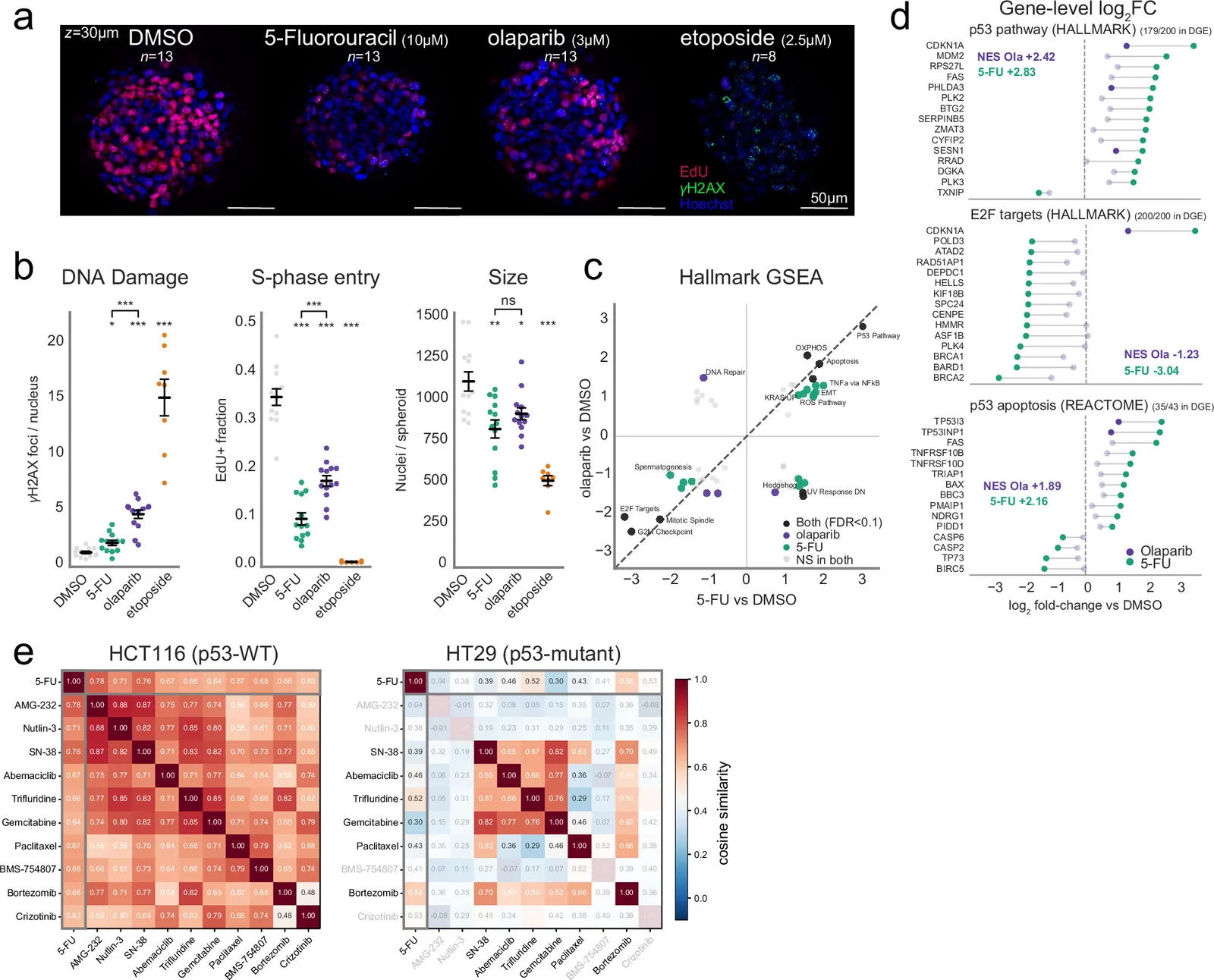
Transcriptional, DNA-damage, and DNA-synthesis readouts characterize the 3D divergence between 5-FU and olaparib. **a** Microscopy images of spheroids exposed to 5-FU (10 µM; *n* = 13; green), olaparib (3 µM; *n* = 13; purple), and etoposide (2.5 µM; *n* = 8; orange), plus a DMSO control (0.1%; *n* = 13; gray). Stained for EdU (red), Hoechst (blue), and γH2AX (green); 12-bit; channels background-subtracted in Fiji across the whole image (rolling ball = 50, 50, 5); display ranges Hoechst 0–2500, γH2AX 11–160, EdU unscaled (0–4095). Scale bars, 50 µm. Images were acquired at 30 × (0.227 µm/pixel) from a 30 µm depth. **b** Quantification of DNA damage (γH2AX foci/nucleus), S-phase entry (fraction of EdU-positive nuclei), and spheroid size (nuclei/spheroid) across the four conditions (n as in (**a**); unit of study, individual spheroid in a separate well); error bars, mean ± SEM. Significance by Kruskal–Wallis followed, where significant, by two-sided Mann–Whitney U; comparisons against DMSO were Bonferroni-corrected, the 5-FU versus olaparib comparison was not. Exact P values versus DMSO for 5-FU, olaparib and etoposide respectively, and for 5-FU versus olaparib: γH2AX foci, 0.0122, 6.2 × 10⁻⁵, 2.9 × 10⁻⁵ and 2.7 × 10⁻⁴; EdU-positive fraction, 4.9 × 10⁻⁵, 6.2 × 10⁻⁵, 4.8 × 10⁻⁴ and 5.9 × 10⁻⁴; nuclei per spheroid, 0.0088, 0.0359, 2.9 × 10⁻⁵ and 0.412 (not significant). **c** Hallmark gene-set enrichment analysis (GSEA): NES for 5-FU (x-axis) versus olaparib (y-axis), colored by agent as in (**a**) or black where significant for both (FDR < 0.1). **d** Per-gene log2FC for the 15 strongest effectors per signature (top to bottom: p53 Hallmark, E2F Hallmark, p53-apoptosis Reactome); colored as in (**a**), opacity indicating significance (FDR < 0.1). **e** Pairwise cosine-similarity maps of the 10 drugs most similar to 5-FU (10 µM) in HCT116 (left; subset of Fig. 4f) and HT29 (right); 5-FU boxed. Source data are provided as a Source Data file.

To understand why olaparib and 5-FU diverged in 3D, we compared their effect on DNA damage, cell-cycle progression, and gene expression. Both drugs interfere with DNA replication, but through different mechanisms: olaparib, a PARP inhibitor, traps PARP1 and causes replication stress^78–80^, whereas 5-FU, a nucleobase analog, depletes nucleotides and disrupts DNA and RNA integrity^81–84^. We therefore measured DNA damage by γH2AX immunostaining and S-phase entry by EdU incorporation in cleared spheroids (Fig. 6a). EdU-positive nuclei were present throughout the spheroid, including its center, indicating that these spheroids lack the proliferating rim and quiescent core typical of larger spheroids^85,86^. Olaparib produced more γH2AX foci than 5-FU (median 4.8 vs. 1.9 foci/nucleus; *p* < 0.001), while 5-FU suppressed EdU incorporation more strongly (median 7.7% vs. 16.3% EdU⁺; *p* < 0.001; Fig. 6b). The lower S-phase entry was therefore not explained by greater DNA damage.

We next compared the transcriptional response to 5-FU and olaparib by bulk RNA-seq of 3D spheroids (Supplementary Fig. 6a). Both drugs enriched p53 and associated apoptotic target genes (Fig. 6c). The p53 effector p21 (CDKN1A) was induced by both and more strongly by 5-FU (log2FC + 3.49, FDR = 8.3 × 10⁻⁷ vs. + 1.35, FDR = 0.025; Fig. 6d), in line with 5-FU’s known activation of p53^87^. E2F-target and G2M-checkpoint signatures were depleted by both drugs, again more strongly by 5-FU, paralleling its stronger suppression of S-phase entry (Fig. 6c, d and Supplementary Fig. 6b). These data point towards engagement of a p53/p21 arrest program that was stronger with 5-FU. Neither the stronger p53 response nor the lower S-phase entry followed γH2AX burden, which was higher with olaparib, consistent with reports that p21 induction can be uncoupled from DNA damage signaling^88^.

With the p53/p21 response established, we returned to the 3D morphological profiles to ask whether 5-FU is placed alongside other drugs that activate p53. Its closest matches are AMG-232^89^, Nutlin-3, and SN-38 (Fig. 6e), which activate p53 through different routes: directly, by inhibiting MDM2, or through DNA damage, by inhibiting topoisomerase-I^90,91^. Moreover, this grouping segregated with p53: in TP53-mutant HT29^92^, the MDM2 inhibitors failed to elicit a phenotype, while 5-FU remained similar to the other antimetabolites. This persistence marks a p53-independent component of 5-FU’s response (Fig. 6e). Altogether, the same 3D morphological readout grouped 5-FU with p53 activators and separated it from olaparib – a distinction present in p53-wild-type HCT116, lost in p53-mutant HT29, and missed by 2D profiling.

## Discussion

Single-cell morphological profiles of 3D spheroids recovered biological relationships between drugs that both spheroid-wide projections and 2D profiles failed to capture. Olaparib and 5-FU, a PARP inhibitor and an antimetabolite that are morphologically near-identical in 2D, produced clearly separated profiles in 3D. We independently corroborated this divergence by EdU, γH2AX, and bulk RNA-seq. These results were made possible by adapting Cell Painting to 3D spheroids at screening scale, retaining compatibility with existing high-content screening infrastructure.

5-FU, a backbone of colorectal cancer regimens, activated the p53/p21 program more strongly than olaparib, yet caused less DNA damage, and grouped with MDM2 inhibitors in a p53-associated manner. This dissociation is consistent with the two drugs engaging p53 through distinct mechanisms: 5-FU via nucleolar stress, stabilizing p53 post-translationally^93^, and olaparib via replication-fork acceleration and DNA damage^94^. A penetration or zonation effect is unlikely, given intracellular olaparib detection^95^, spheroid size^85,86^, and ongoing proliferation (Fig. 6a, b). How distinct stresses converge on p53 is not fully understood^96^. Because intracellular signaling itself is reshaped in 3D^18,24,26^, we propose the divergence reflects the 3D format altering how each drug’s particular mix of DNA damage, replication stress, and nucleolar stress is processed into a p53/p21 response. The divergence was detectable in single-cell profiles only, not in spheroid-wide projections.

Resolving single cells inside dense 3D structures at the screening scale is a central technical challenge. Dedicated light-sheet platforms achieve volumetric single-cell imaging at high spatial resolution with minimal bleaching, but depend on specialized hardware^97–99^. More conventional high-content systems rely on projection-based readouts that sacrifice single-cell detail. Our method sits in between: on a standard high-content spinning-disk confocal, an open-source detection step localizes spheroids in z, providing the per-section depth metadata our normalization uses to mitigate signal attenuation.

In designing this workflow, we reasoned that per-plane 2D detection captures morphologies representative of the 3D structure, obviating full 3D segmentation. At our *z*-sampling, each cell was detected three- to fourfold, yielding 2D instances rather than a unique cell count; this redundancy left profile robustness intact at coarser *z*-steps, up to 10 µm. Staying per-plane also kept data volumes manageable: 3D segmentation would require near-isotropic voxels our acquisition does not provide (*z* = 5 µm vs. *xy* = 0.227 µm), and resampling our stacks would inflate them roughly 20-fold. Pushing to finer axial sampling on our spinning-disk confocal brought limited benefit: a 1.5 µm *z*-step did not improve reproducibility (Supplementary Fig. 4b) and added bleaching across the many extra planes (Supplementary Fig. 4g, h).

For groups adopting this workflow, these choices translate into concrete defaults: 30 × magnification with 20–40% glycerol/40% fructose clearing, a coarse axial step (5–10 µm), and moderate seeding ( ~ 150 µm spheroid diameter), while avoiding 40× and over-large spheroids. These are the conditions under which single-cell resolution was recoverable on our standard high-content hardware. Our approach also has limitations. Effect-size comparisons between 2D and 3D, such as the grit-score differences, are sensitive to technical factors: depth-dependent attenuation and segmentation variability across sections could both lower grit scores independently of biology. Our per-section normalization mitigates attenuation but collapses single-cell information and assumes minimal biological variation with depth. Compounds that strongly alter spheroid optics (necrosis, vacuolization) may also attenuate light differently from the controls they are normalized against, leaving possible residual bias. Our biological interpretation carries its own caveats: the dissociation of p53 response from DNA-damage burden rests on a single drug pair, and the attribution of the morphological grouping to p53 relies chiefly on the MDM2 inhibitors, as the HCT116–HT29 comparison differs in more than p53 status.

3D Cell Painting could extend morphological profiling to culture models that are difficult to capture in 2D, such as liver culture models, which tend to flatten and lose some of their function in 2D^100,101^, organoids, or models that co-culture immune cells^102^. Integrating more labels such as antibody-based identity markers would also be a relevant next step^103^. 3D culture remains less standardized than 2D, with diverse methods and terminology^104–106^, but the standard ULA-plate format used here keeps single-cell 3D profiling compatible with existing high-throughput workflows. Our approach is particularly suited to compact, solid 3D models lacking a lumen, which are the structures where projection-based readouts may lose the most single-cell information.

## Methods

### Cell lines

Two human colorectal cancer cell lines were used: HCT116 (RRID:CVCL_0291; colorectal carcinoma, adult male donor; originally ATCC CCL-247) and HT29 (RRID:CVCL_0320; colorectal adenocarcinoma, female donor; originally ATCC HTB-38). Both lines were provided by T. Visnes (SINTEF Industry, Trondheim, Norway) and were routinely tested for mycoplasma using a luminescence-based Lonza Mycoplasma kit (Lonza™ LT07-418), testing negative. In addition, both cell lines were authenticated by short tandem repeat (STR) profiling (Eurofins Genomics GmbH). The profiles matched the reference profiles for HCT116 and HT29. Neither line appears on the register of misidentified cell lines maintained by the International Cell Line Authentication Committee (ICLAC). Both cell lines were cultured in RPMI 1640 medium (Gibco 31870) supplemented with FBS (10%; Sigma F7524), Penicillin-Streptomycin (100 U/ml; Gibco 15140-122), and GlutaMax (1 ×; Gibco A1286001), kept at 37 °C in a humidified atmosphere at 5% CO_2_ and passaged when confluency reached 80–90% using Trypsin-EDTA (0.25%; Gibco 15400054) for dissociation. Cells used in experiments never exceeded passage 10 after thawing. Sex was not considered as a variable in the study design.

### Clearing solution

Prior to the experiment, we prepared 100 ml of clearing solution by mixing 40 ml glycerol (40% vol/vol), 40 g of fructose (40%; wt/vol; 2.2202 M), and 32 ml of dH2O on a magnetic stirrer. This solution had a refractive index of 1.451–1.452 at RT as measured with a refractometer.

### Compound library and compound spotting

A clinical library encompassing 52 small molecules was manually curated by relevance for (colorectal) cancer therapy and mechanism of action. Actual clinical status was retrieved from the drug repurposing hub^107^. Available drugs were sourced from SelleckChem as a Cherry Pick Library. The remaining drugs AMG-232 (TA9H93ED7611; Sigma) and 5Z-7-OXOZEAENOL (O9890; Sigma) were purchased separately from Sigma-Aldrich. Four concentrations (10 µM, 3 µM, 1 µM, and 0.3 µM) per compound were screened. Compound libraries were stored in an airtight container at − 20 °C before use. We included two phenotypic reference compounds with a known distinctive phenotype^54^: etoposide (E1383; Sigma), fenbendazole (F5396; Sigma). Furthermore, staurosporine (19–123; Sigma) was included as cytotoxic control^108^, D-Sorbitol (S1876) as negative control, and DMSO (D2438) as compound vehicle (purchased from Sigma Aldrich). Supplier, catalog number, and CAS registry number for all 52 library compounds are provided in Supplementary Data 1; all compounds were ≥ 98% pure according to the suppliers’ certificates of analysis and were used as received.

To reduce the impact of plate effects, we both excluded outer wells and designed effective microwell layouts using constraint programming with PLAID – Plate Layouts using Artificial Intelligence Design^55^. We included two biological and two technical replicates, and each plate had 20 interspersed DMSO controls. We spotted drugs onto Corning® 384-well microplates (CLS 3656) using the I.DOT (I.DOT; Dispendix, Stuttgart, Germany) with the I.DOT PURE S Plate 100 (D16110021800). When plates were not used immediately, they were sealed and stored at − 20 °C. Before use, we first dissolved the drugs in 10 µl cell medium (4 ×). Afterwards we centrifuged (50 × *g*; 20 s) and incubated the plates on a shaker at RT (20 min). For 2D experiments, drugs were directly spotted onto PhenoPlate™ 384-well microplates (Revvity; 6057302).

### 3D Spheroid cell painting

#### Cell seeding and compound treatment

We seeded cells using the ViaFlo 384 (Integra) in 30 µl cell medium on 384-well ULA spheroid microplates (Corning; 3830). Cells were seeded at 270 cells/well (HCT116; p + 10) or 300 cells/well (HT29; p + 8) in all wells. To support spheroid aggregation, we centrifuged (120 × *g*; 2 min) and incubated the plates (24 h; 37 °C; 5% CO_2_). Afterwards, we exposed the spheroids by transferring 10 µl of drugs in cell medium using the ViaFlo for a final volume of 40 µl.

#### The cell painting assay

Following 48 h compound treatment, we performed an adapted version of the Cell Painting Assay^61,109^ implemented wash steps with the Biotek 405 LS and dispense steps with the Biotek MultiFlo FX: We washed the cells once with 60 µl 1 × PBS (Gibco™ 18912014), incubated 4 min, and aspirated, leaving 20 µl in the well. Then, we fixed with 60 µl 4% PFA (Histolab; 02176) for 1 h at room temperature. We washed twice (60 µl, 5 min wait, 1 × PBS, leaving 20 µl) and permeabilized with 60 µl Triton X-100 0.2% (final well concentration; Thermo Scientific 85111) for 1 h at room temperature, washed once (60 µl, 1 × PBS, leaving 10 µl), and finally stained with 20 µl stain mixture, including Hoechst 33342, SYTO 14, phalloidin, concanavalin A-conjugate, wheat germ agglutinin (WGA)-conjugate, and MitoTracker Deep Red (see Supplementary Data 3) overnight (18 h) at 4 °C. The next day, we washed twice (60 µl, 2.5 min wait, 1 × PBS, leaving 10 µl) and dispensed 70 µl of clearing solution. We sealed and kept plates for 72 h at 4 °C, and acclimatized to RT for 1 h prior to imaging.

### 3D Image acquisition

We imaged the plates in the Nikon ECLIPSE Ti2-E inverted microscope using a 20× objective (PLAN APO λD 20 × OFN25 DIC N2, wd = 0.8 mm, NA = 0.8) combined with an additional 1.5 × zoom, to create a total zoom of 30 × (res = 0.227 µm/pixel). The microscope was fitted with a Celesta light engine and 50 µm spinning disk pinhole (spacing = 250 µm). Images were captured on a Prism BSI Express sCMOS camera (Teledyne Photometrics) using NIS-Elements 5.47.07 (Nikon). Each channel was imaged with the filter settings and exposure times described in Supplementary Data 3.

### Spheroid detection method

To detect the spheroids, we used a custom protocol in Nikon JOBS and GA3. In summary, we scanned every well at 4 × in the SYTO channel (widefield, binning = 2x, exposure time = 5 ms, lase*r* = 0.42). By thresholding, we automatically estimated the position of each spheroid (threshold = 1424). At each measured location, the entire spheroid was first scanned at 30× in SYTO (range = 550 µm, ste*p* = 10 µm, confocal = 50 µm, exposure time = 50 ms, lase*r* = 0.42). From this *z*-stack we would calculate the z-differential of the FocusScore – or normalized variance^14,52^. A higher z-differential, or in other words, a sharp change in Focus, would indicate the start of a spheroid. At this measured z location, we started imaging the spheroid in 5 channels (Supplementary Data 3), with 5 µm spacing across 12 optical sections. Images were saved as 16-bit ome.tiff files per channel (1048 × 1048). Direct comparison against existing spheroid-detection methods was not possible, as no publicly available implementations exist to our knowledge; an example dataset has therefore been deposited at the BioImage Archive (accession S-BIAD2254) to enable future comparisons.

### 2D Monolayer cell painting

For 2D experiments, we first dispensed 20 µl of cell medium in 384-well PhenoPlates and incubated them at room temperature for 20 min, followed by dispensing 20 µl cell mixture using the Biotek MultiFlo FX (HCT116, 2600 cells/well, p + 8; HT29, 2000 cells/well, p + 6).

Finally, the plates were kept in an incubator (48 h; 37 °C; 5% CO_2_).

For the 2D Cell Painting assay, conditions matched the spheroid protocol except that MitoTracker was applied as a live pre-fixation stain, all reagent volumes were 80 µl, fixation and permeabilization used shorter incubations (4% PFA, 20 min; 0.1% Triton X-100, 20 min), the stain mixture was applied for 20 min at room temperature, and no clearing step was used (Supplementary Table 2). Compound concentrations were 10 µM, 3 µM, 1 µM, 0.3 µM, and 0.1 µM.

We imaged the plates at 20 × (res = 0.34 µm/pixel) by using Nikon’s perfect focus system (PFS) to capture nine sites per well (2048 × 2048), 16-bit, in five fluorescent channels (Supplementary Data 3).

### Second-site validation

The 3D Cell Painting workflow was reproduced at a second site (AstraZeneca) on a Yokogawa CV8000 50 µm spinning-disk confocal microscope (20 × LWD dry, NA 0.45 or water-immersion, NA 1.0; Hamamatsu sCMOS cameras; Yokogawa BTS software), using six compounds (berberine chloride, etoposide, fluphenazine, fenbendazole, nocodazole, and sorbitol) across a 10-point dose–response (Supplementary Table 1). Each channel was imaged with the filter settings and exposure times described in Supplementary Data 3. Since we did not use a specific spheroid detection algorithm in Z, the entire column was captured, 250 µm above and 100 µm below the calibrated reference point with 5 µm spacing. Images were saved as 16-bit ome.tiff files per channel (1994 × 1994).

### Feature extraction and preprocessing

We processed images in CellProfiler^14^ with the Cellpose v2.0 plugin^53^, which we retrained per cell line on representative nuclei (Hoechst) and cell (PHAandWGA) images spanning different drugs and, for 3D, different optical sections. Training and detection were run on raw images; for the 3D models, we additionally subtracted background in Fiji to ease manual labeling. We then extracted features per object, and preprocessed them in Pycytominer^13^: well-aggregation, per-plate standardization to negative controls (DMSO; features *z*-scored on the DMSO mean and standard deviation), clipping of the standardized feature values at ± 40, and feature selection (blocklist, variance, and correlation thresholds; NaN removal). Pipelines and retrained models are available (Code availability); full parameters are in Supplementary Table 3. The three pipelines differed as follows:

#### 3D single cells (scAgg)

We extracted features per cell on each optical section, aggregated them per section (median), and standardized to DMSO controls both per plate and per section to correct for depth-dependent signal degradation, before aggregating per well (median). No illumination correction was applied.

#### Maximum-intensity projections (MIP)

We generated spheroid-wide maximum-intensity projections using Cell Profiler and applied per-channel quality control on the PowerLogLogSlope, flagging images above the 93rd percentile and excluding those flagged in more than one channel. We then segmented each spheroid and extracted features per spheroid, removing out-of-focus spheroids before preprocessing.

#### 2D monolayer

We applied illumination correction and outlier-based QC, and aggregated features by mean over sites, then median over wells.

#### Second-site validation (Astrazeneca)

Spheroid boundaries were detected post hoc using SphereDetect (offset = -2, fmi*n* = 250, downsample = 4), where *offset* adjusts the detected start position relative to the peak differential, and *fmin* refers to the minimum value for the focus score at the maximum change. The newly calculated Z labels were then used to process features. We calculated median pairwise Spearman correlations for each perturbation (compound × concentration). When only one or fewer technical replicates remained due to missing data, the perturbation was assigned a value of 0. Before computing the UMAPs, low concentrations (0.0316, 0.0100, 0.00317, 0.00100, and 0.000316 µM) were filtered out from the data. UMAPs were calculated with the following settings: (1) unlabeled [n_neighbors = 6, *random_state* = 42, metric = ‘euclidean’, *min_dist* = 0.01, spread = 2] and 2) labeled [n_neighbors = 10, *random*_*state* = 42, metric = ‘euclidean’, *min*_*dist* = 0.1, spread = 5].

### Data analysis and visualization

We implemented data analysis in Jupyter notebooks, applied consistently for each data type (MIP, scAgg, or 2D).

#### Perturbation filtering

Grit scores, which quantify both the strength and robustness of morphological perturbations^58^, were calculated by using the cytominer-eval package^60^. Morphological profiles were filtered to retain perturbations with grit > 1.96 (corresponding to the two-sided 95% critical value of the null distribution).

#### Technical-replicate reproducibility

Technical-replicate reproducibility was quantified per compound as the median pairwise Pearson correlation between replicate profiles relative to a matched non-replicate (null) distribution, following^61^ across acquisition parameters (*z*-plane subsampling, *z*-sampling density, spheroid seeding density, clearing, and magnification). Within each condition, replicate correlations above the null were tested by two-sided Mann–Whitney U. Between conditions, null-subtracted correlations were compared by a bootstrap over compounds: pairs were called equivalent if the 90% CI fell within ± 0.10 r (≡) or different if the 95% CI excluded 0 (asterisk). Comparisons are exploratory, without multiple-comparison correction.

#### Dimensionality reduction and clustering

UMAPs were generated using the UMAP-learn package^66^. Labeled UMAPs were generated using onehot encoding. Unsupervised UMAPs were generated with the following settings: n_components = 2, spread = 5, n_neighbors = 200, min_dist = 0.1, random_state = 42, metric = ‘cosine’. We used the scikit-learn package to cluster spheroids by k-means on the 2D UMAP embedding (*k *= number of pathways) and to score cluster–pathway agreement with the Adjusted Rand Index (ARI) and Normalized Mutual Information (NMI); the silhouette score (SC) was reported descriptively. One-sided *p*-values for ARI and NMI were obtained by permuting the pathway labels 1000 times (random_state = 42) with the clusters held fixed; SC was not permutation-tested.

#### Similarity analysis

For each compound passing the grit threshold (grit > 1.96), a consensus profile was computed as the median across its treatment wells. Pairwise cosine similarities between these consensus profiles were assembled into a compound x compound similarity matrix and visualized using the seaborn *clustermap* function. Rows and columns were ordered by hierarchical clustering with Ward linkage applied to Euclidean distances between similarity profiles. The number of clusters was chosen by sweeping *k* = 2–8 and maximizing the average silhouette score, with the adjusted Rand index against annotated target pathways reported at each k.

For the 2D-versus-3D comparison, cosine similarity was computed between every pair of compounds separately for the 2D and 3D profiles, and pairs in both modalities were matched. For each pair, we recorded the signed between-format difference (3D − 2D) and the higher of the two similarities. Pairs whose difference fell in the extreme 5% of the difference distribution across all matched pairs were flagged as divergent; among these pairs, whose higher similarity exceeded 0.5 in at least one format were labeled in the figure.

#### Visualization

For visualization, we subtracted the background in Fiji using the rolling-ball algorithm, applied uniformly to the whole image of each channel. No gamma or other nonlinear adjustment was applied at any stage. All displayed images use linear LUTs; per-figure display ranges and background-subtraction radii are given in the legends. To display orthogonal slices of the spheroids, we made the data isotropic using Clij2^110^.

### Acquisition-parameter analysis

To define the method’s operating range, we varied key acquisition parameters one at a time, holding other settings at their standard values, and assessed each condition for technical-replicate reproducibility (median pairwise replicate correlation against a matched non-replicate null; see Data analysis and visualization). All experiments used HCT116 spheroids (treatment wells only). Comparisons used a common perturbation set and a matched number of replicates, so that differences reflect the acquisition parameter rather than panel composition or replicate count.

#### Magnification

We compared 10 ×, 30 × (20 × objective with 1.5 × zoom; our standard) and 40 × (6 compounds, N-matched).

#### Axial sampling

We compared sparse acquisition (5 µm spacing, 12-plane stacks) against finer near-Nyquist sampling (1.5 µm spacing, 44 planes). Within the sparse regime, we computationally subsampled the acquired stack: single mid-stack planes (z2, z7, z11) and 3-, 6-, and 9-plane subsets against the full 12 planes (50 compounds). For the finer regime, we compared the full 44-plane stack against a 14-plane subsample of the same grid, separating z-coverage from plane spacing.

#### Clearing

We compared spheroids imaged with and without optical clearing, as well as a reduced glycerol condition (20% v/v) on two measures: replicate reproducibility and cell-detection depth (cells segmented as a function of depth, with planes converted to µm using the per-condition *z*-step: 5 µm for cleared/uncleared, 1.5 µm for Nyquist stacks).

#### Seeding density / spheroid size

We varied seeding density, and thereby spheroid size, up to 810 cells/well (original, 2 ×, and 3 × seeding).

### Signal intensity across *z*-step sampling

Photobleaching with depth was assessed in DMSO control wells of two HCT116 acquisitions (5 µm and 1.5 µm *z*-steps). For each stain, per-well intensity profiles were normalized to the most-superficial slice (I/I₀), interpolated onto a common depth grid, and summarized across wells by the median with a bootstrap 95% CI. The two *z*-step curves were compared per stain with a cluster-based permutation test^111^: at each depth bin a two-sample Welch t was computed between conditions across wells, and contiguous bins exceeding a two-sided *p* < 0.05 threshold were grouped into clusters, each summarized by the sum of its *t*-values and tested against a null distribution from 2000 whole-well permutations. Cluster-level *p*-values were FDR-corrected, and clusters significant at *p* < 0.05 are reported. The same test was applied to per-slice detected-cell counts, comparing each clearing condition to the 20/40% reference. Analyses used numpy and scipy.stats.

### Duplication estimates for section-wise segmentation

Because we segmented each optical section independently, a cell spanning multiple sections is detected on each, so raw counts reflect 2D segmentation object instances per spheroid rather than unique cells. To estimate this redundancy, we linked 2D segmentation instances across adjacent *z*-planes: for each object in plane z, we linked the object in plane z + 1 with the highest intersection-over-union when the overlap exceeded 0.3, extending each track greedily until no qualifying successor remained. We retained objects without successors as single-plane tracks. Each track represents one putative cell, and its length the number of redundant detections. We summarized detections per cell per well and used the resulting duplication factor (Fig. 2e) to convert 2D segmentation object instances to approximate unique cell numbers per spheroid.

### Expert annotations

To quantify the accuracy of Cellpose segmentation and assess how segmentation error propagates to morphological features, an expert-annotated reference set was generated for HCT116 and HT29 spheroids exposed to DMSO, binimetinib, abemaciclib and SN-38. These compounds were selected to cover the unperturbed control phenotype and three distinct drug-induced morphologies (compact cytoplasm, enlarged cells, and vacuole-rich cells). Nuclei and cell boundaries were manually annotated in the cellposeGUI^53^ on 6 optical sections per condition, sampled across shallow, mid and deep regions of the spheroid, yielding 8492 annotated nuclei and 6429 annotated cells. IoU was computed per-pixel between Cellpose and expert masks within each *z*-section. To assess propagation to features, morphological profiles were extracted from Cellpose and expert masks using identical CellProfiler pipelines, and Pearson correlations were computed between matched per-object feature profiles.

### EdU and γH2AX assay: staining, imaging, and analysis

HCT116 (p + 10) spheroids (270 cells/well, grown 24 h) were treated for 48 h with 5-FU (10 µM), olaparib (3 µM), etoposide (2.5 µM), or 0.1% DMSO vehicle (*n* = 13 spheroids per condition, except *n* = 8 for etoposide), pulsed with EdU (5 µM, 2 h), then fixed and stained for EdU (Click-iT Alexa Fluor 647), phospho-γH2AX (Ser139), and Hoechst, and cleared before imaging (reagents, dilutions, and wash steps in Supplementary Table 4).

Spheroids were imaged on a spinning-disk confocal at 30× (0.227 µm/pixel) using the acquisition workflow described above (4 × scan for X–Y position, Z-scan for the first optical section, then multichannel imaging), with custom spheroid detection scanning in the Hoechst channel. Three channels (Hoechst/405, γH2AX/488, EdU/647; filters in Supplementary Data 3) were acquired at 12-bit across 10 optical sections.

Nuclei were segmented (CellProfiler with the Cellpose nuclear model) and γH2AX foci counted per nucleus; EdU-positive (S-phase) cells were called by a per-spheroid Otsu threshold on log-transformed nuclear EdU intensity. Per-spheroid metrics (nucleus count, EdU-positive fraction, γH2AX foci) were aggregated over *z*-planes and compared by Kruskal–Wallis; where the omnibus test was significant (*p* < 0.05), pairwise comparisons against DMSO used Mann–Whitney U with Bonferroni correction; full parameters are in Supplementary Table 4.

### Bulk RNA-seq

Two HCT116 (p + 9) vials were each seeded across two 384-well ULA plates (270 cells/well; 128 wells/condition/plate), giving 2 biological × 2 technical replicates per condition. Plates were centrifuged (120 × *g*, 2 min), grown for 24 h, and treated with 5-FU (10 µM), olaparib (3 µM), or 0.1% DMSO. After 48 h, spheroids were harvested by plate inversion (trays rinsed between replicates), and the 128 wells per condition per plate were pooled in PBS, pelleted (200 × *g*, 5 min), and lysed, yielding 12 samples (3 conditions × 4 replicates). Total RNA was extracted with the RNAqueous-Micro kit (Thermo Fisher, AM1931; including on-column gDNA removal), eluted in IDTE buffer (10 mM Tris, 0.1 mM EDTA, pH 7.5; IDT), and quantified by NanoDrop (Thermo Fisher). RNA was then stabilized with SEQguard Dino Preserve (Genovis, PS-SDP-024) for ambient shipping to Plasmidsaurus, where library preparation, 3′-end counting on an Illumina NovaSeq X Plus ( ~ 10 million deduplicated reads/sample), and primary analysis were performed. One DMSO replicate was excluded for low read count ( < ~3 million deduplicated reads). Gene-level differential expression (edgePython v0.2.5) and functional pathway enrichment (GSEApy v0.12; reported as normalized enrichment score, NES, at false discovery rate, FDR < 0.1) against gene sets from MSigDB v2026.1 human symbol GMT files — the Hallmark collection (h.all.v2026.1.Hs.symbols.gmt, 50 sets) and the C2 curated collection (c2.all.v2026.1.Hs.symbols.gmt, 7670 sets) used the provider’s built-in tools with all replicates as separate samples; output tables were used directly to generate figures.

### Statistics and reproducibility

All spheroid Cell Painting data (Figs. 2–5, Fig. 6e, Supplementary Fig. 3 and Supplementary Fig. 5) derive from a single experimental run of six 384-well plates, with two biological – cell stocks thawed separately - and two technical replicates per condition and 20 interspersed DMSO controls per plate. The protocol-robustness comparisons (Supplementary Fig. 4a–h) and the cross-site validation at AstraZeneca (Supplementary Fig. 4i–k) were acquired in separate runs. Reported replicate correlations reflect within-run reproducibility. The 2D dataset (Fig. 5a–d) came from a separate four-plate run with the same replicate structure (2 biological x 2 technical).

From Fig. 4 onwards, analyses were restricted to perturbations exceeding grit > 1.96.

The EdU/γH2AX experiment (Fig. 6a, b) was performed once. Each condition comprised 13 spheroids in separate wells (8 for etoposide), analyzed as technical replicates. For Fig. 6b, the three comparisons against DMSO were treated as one family and Bonferroni-corrected within each metric; the single 5-FU versus olaparib comparison was not corrected.

RNA-seq (Fig. 6c, d and Supplementary Fig. 6) used two independently thawed HCT116 vials, each seeded across two plates, giving two biological and two technical replicates per condition (*n* = 4 per drug; *n* = 3 for DMSO after exclusion of one low-read-count sample). Libraries were prepared and sequenced in a single run. No statistical method was used to predetermine sample size.

### Reporting summary

Further information on research design is available in the Nature Portfolio Reporting Summary linked to this article.

## Supplementary information

Supplementary Information

Description of Additional Supplementary Files

Dataset 1

Dataset 2

Dataset 3

Reporting Summary

Transparent Peer Review file

## Source data

Source Data

## Data Availability

The imaging data generated in this study have been deposited in the BioImage Archive database under accession code S-BIAD2254, a repository previously described^112^. The bulk RNA-seq data generated in this study have been deposited in the NCBI Gene Expression Omnibus database under accession code GSE343958. The BioImage Archive deposit comprises raw 16-bit OME-TIFF images, per-compartment single-cell CellProfiler feature tables (cells, cytoplasm, and nuclei), and the corresponding Cellpose segmentation masks, organized per acquisition plate; a separate detection demonstrator dataset is also included. Folder structure and file naming are documented in the deposit’s README. The source data underlying Figs. 2–6 and Supplementary Figs. 1–6 are provided in the Source Data file. All data generated in this study are publicly available without restriction. Source data are provided in this paper.

## Code availability

Code to reproduce all processing and analysis from these tables is available at https://github.com/pharmbio/colopaint3D, archived at Zenodo (https://doi.org/10.5281/zenodo.22013751)^113^. The SphereDetect autofocus and spheroid detection routine is available at https://github.com/pharmbio/SphereDetect, archived at Zenodo (https://doi.org/10.5281/zenodo.22011585)^114^. All code is released under the MIT license.
