# Supplementary material for: High-content morphological profiling by Cell Painting in 3D spheroids
Source: Nat Commun. 2026 Oct 21;17:10790. doi: 10.1038/s41467-026-78596-5 (PMC13686983; doi:10.1038/s41467-026-78596-5)
Supplement: Supplementary file 1 [file 41467_2026_78596_MOESM1_ESM.pdf]

Supplementary Information

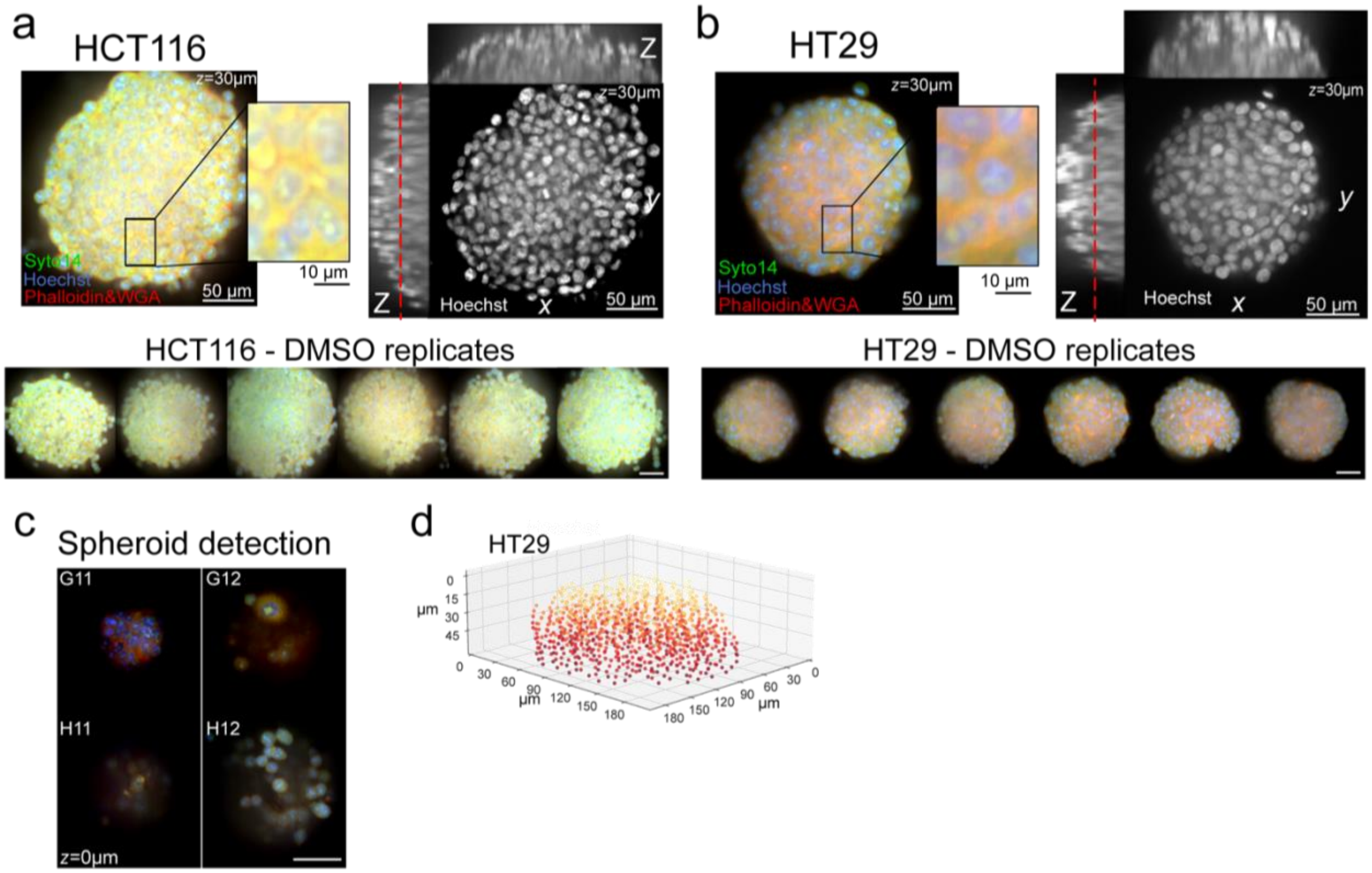

Supplementary Figure 1: Examples of negative controls. (a; left) Composite image of a HCT116 spheroid at 30 µm depth depicting RNA and cytoplasm with (SYTO 14; green), nuclei with (Hoechst; blue) and cytoskeleton plus Golgi (Phalloidin&WGA; red). Individual cells are seen in the inset. (right) Displayed are orthogonal views of the same spheroid in the Hoechst channel. These views consist of 12 made-isotropic slices (ranging from 0 to 60 µm, with 5 µm spacing). A dashed red line indicates the position of a (left). (bottom) Six representative examples of HCT116 spheroids treated with DMSO. (b) an identical panel as A, but for HT29. (c) Representative images of detected spheroids. They represent the first optical sections as found by the detection algorithm. (d) The center positions of detected cells in HT29 spheroids exposed to 0.1% DMSO. Scale bars are 50 µm. Spheroids were imaged at 30× (0.227 µm/pixel) from a 30 µm depth. Source data are provided as a Source Data file.

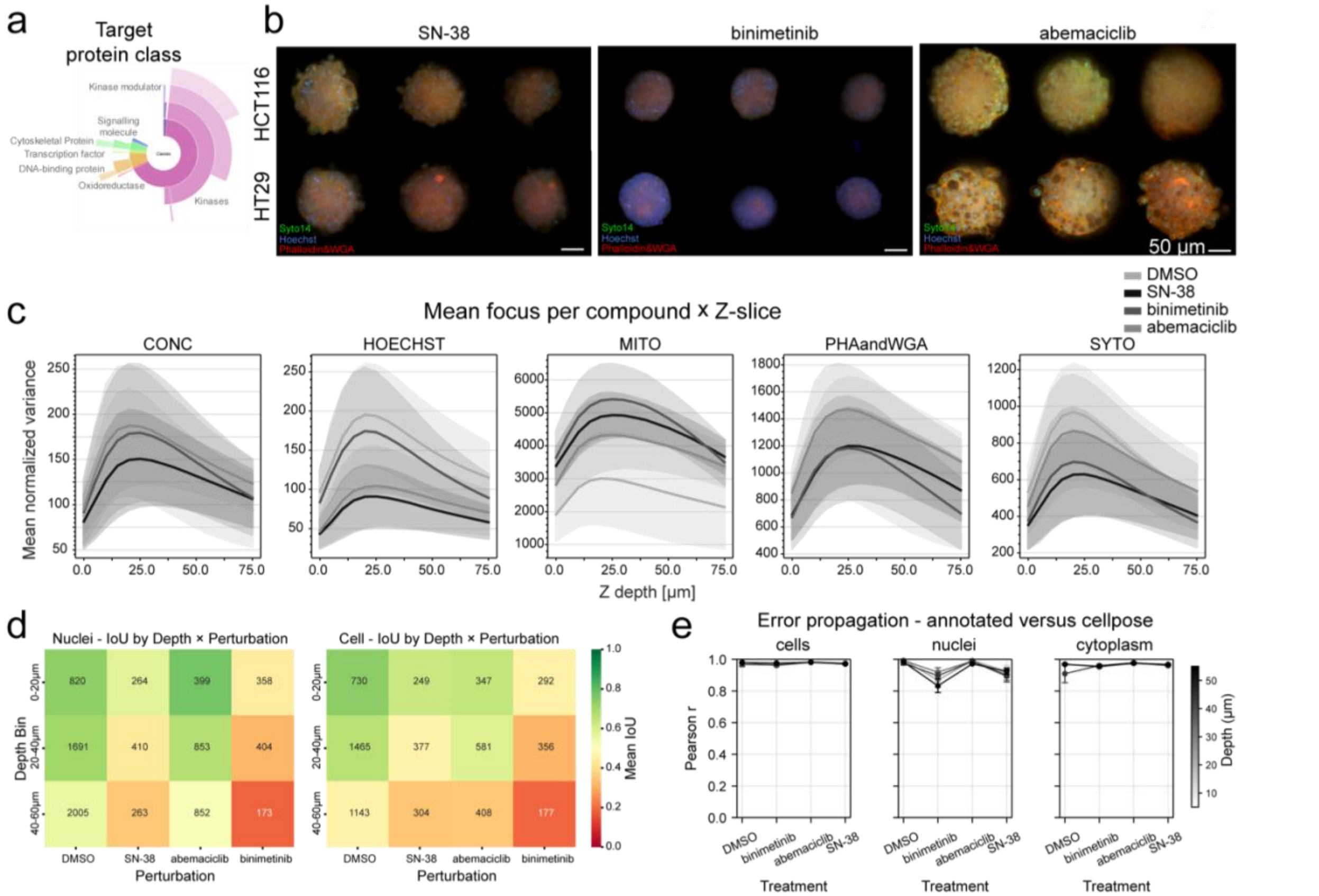

Supplementary Figure 2: Examples of treated spheroids. (a) A diagram depicting the various targets in our drug library. The figure was created using the clue.io website. (b) Examples of HCT116 (top) and HT29 (bottom) single sections at 30 µm depth for the compounds SN-38 (0.03 µM), binimetinib (10 µM), abemaciclib (3.0 µM), and DMSO (0.1%). SYTO 14 (RNA) is shown in green, Hoechst (nuclei) is shown in blue, and MitoTracker (mitochondria) is shown in red. (c) Line graphs indicating the Mean normalized variance as a function of depth, separated by drug and channel (n = 114 wells DMSO, 27 binimetinib, 30 abemaciclib, 26 SN-38). Error shading is ± 1 SD. (d) A heatmap depicting segmentation accuracy of Cellpose against expert annotations, shown as (Intersection of Union) IoU stratified by channel (nuclei, cell) and imaging depth (shallow, mid, deep), pooled across both cell lines (HCT116, HT29), for spheroids exposed to DMSO, binimetinib, abemaciclib and SN-38 (n = 12 images per condition — object type × drug × depth — comprising 6 HCT116 and 6 HT29 images; 8,492 nuclei and 6,429 cells annotated in total). (e) Pearson correlation between per-object morphological feature profiles extracted from Cellpose masks versus expert masks, stratified by drug and channel (n = 36 well × plane measurements per compartment and treatment, except binimetinib (n = 33), from 22 wells across 4 plates). All scale bars are 50 µm. Spheroids were imaged at 30× (0.227 µm/pixel) from a 30 µm depth. Source data are provided as a Source Data file.

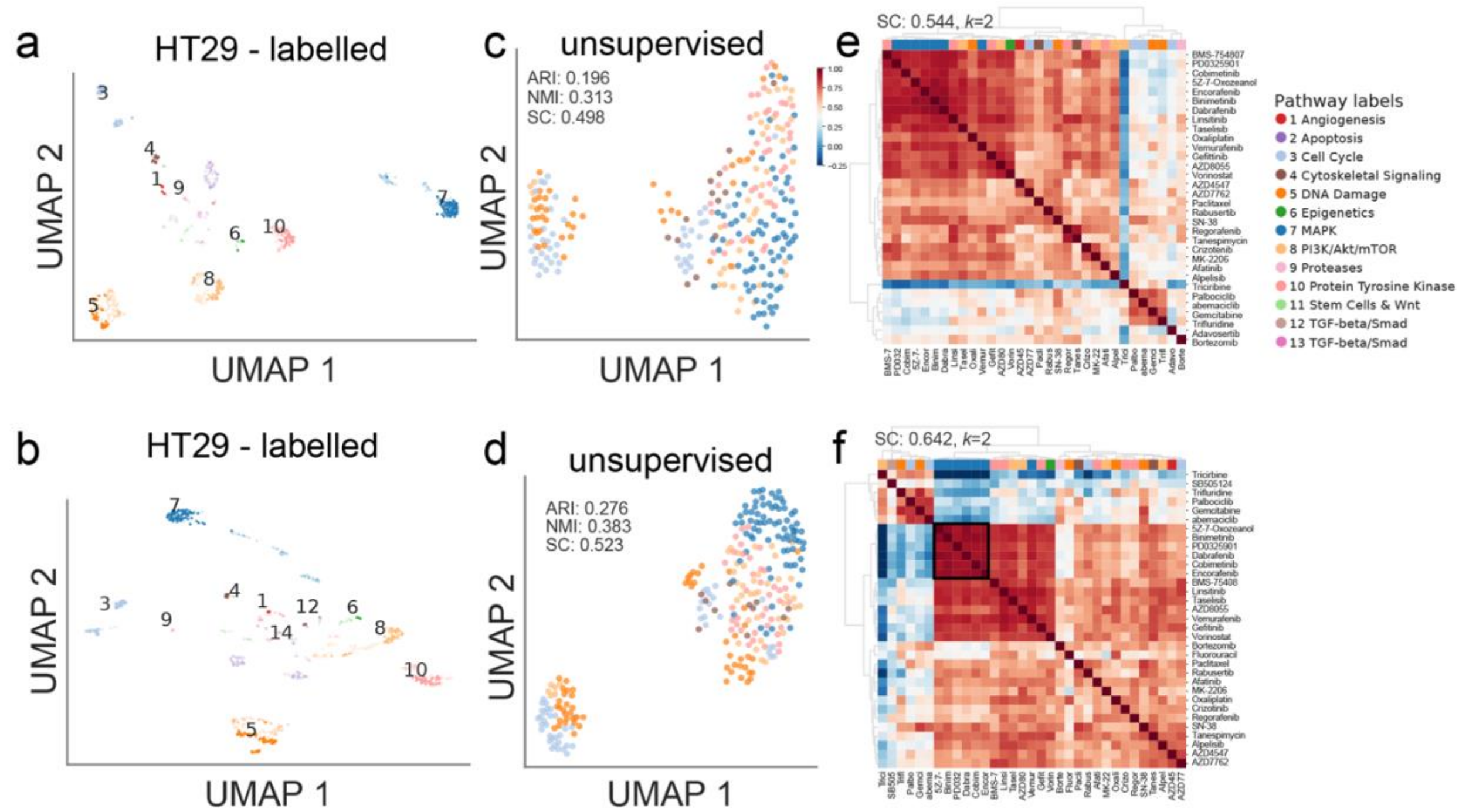

Supplementary Figure 3: Single-cell morphological profiles facilitate the detection of biologically relevant clusters. (a) Semi-supervised UMAPs of MIP-based and (b) scAgg morphological profiles. (c) Unsupervised UMAPs of MIP-based and scAgg (d) morphological profiles. Pathway enrichment scores (ARI, NMI) and cluster cohesion (SC) are provided in the plot. (e) Heatmap displaying hierarchical clusters of pairwise cosine similarities for MIP-based and (f) scAgg profiles. Ward linkage was used to create the dendrogram; the optimal number of clusters (*k*=2) was selected by maximizing the average silhouette score (MIP: SC=0.544, scAgg=0.642). In all panels, pathways are color-coded using the same colormap. All analyses based on HT29 spheroid perturbations with a grit score > 1.96. Source data are provided as a Source Data file.

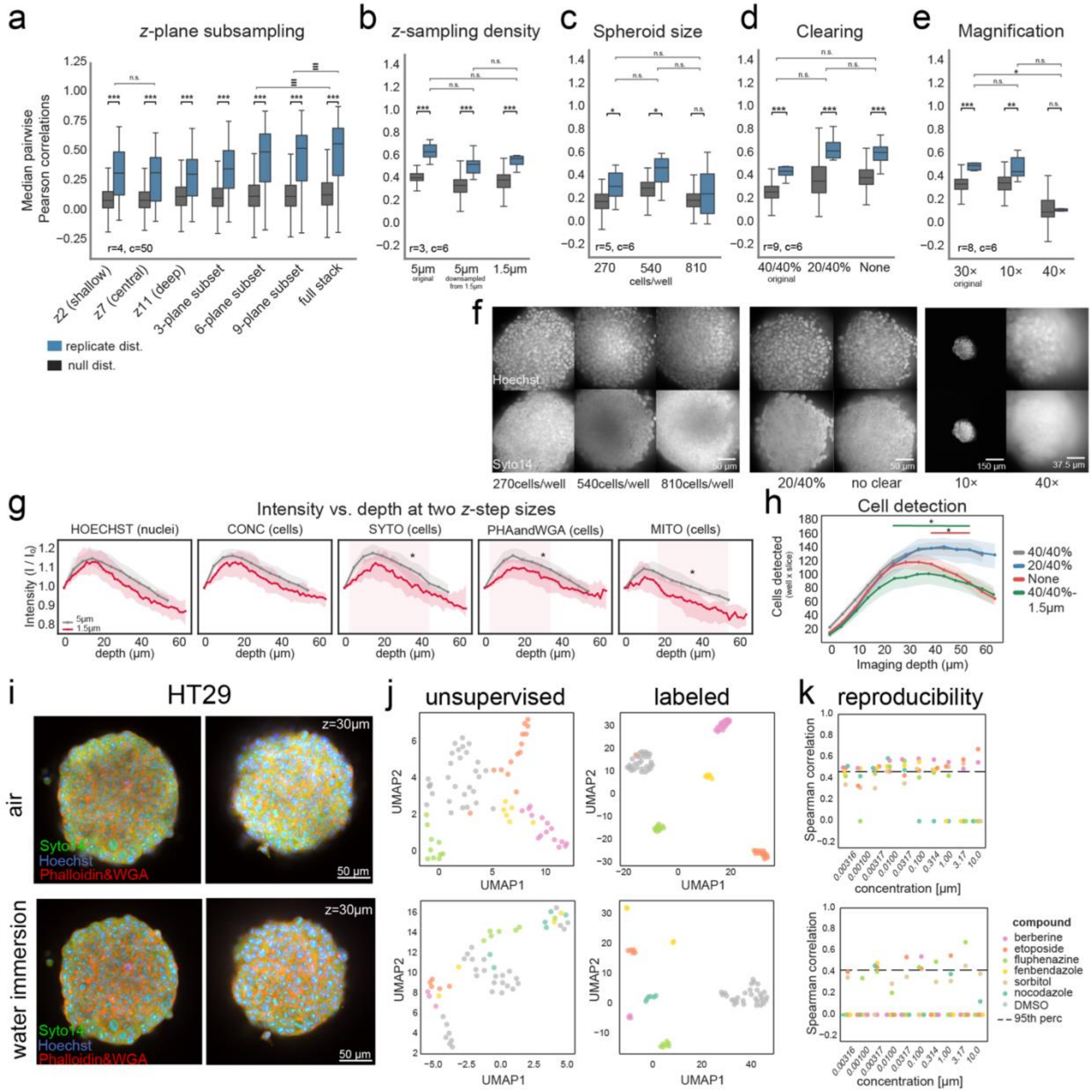

Supplementary Figure 4: Reproducibility of morphological profiles across acquisition conditions. (a-e) Per condition, box plots of median pairwise Pearson correlations between replicate profiles (blue) and a matched non-replicate null (black). *Within* each condition, replicate signal above its own null was tested by two-sided Mann–Whitney U. *Between* conditions, per-compound replicate correlations (null-subtracted) were compared by a bootstrap over compounds: conditions were deemed equivalent if the 90% CI fell within ± 0.10 (Pearson r) and different if the 95% CI excluded 0. Tested parameters are: (a) *z*-plane subsampling (n = 50 compounds, 4 replicates per compound); only the marked comparisons were not significant. (b) z-sampling density (n = 6 compounds, 3 replicates per compound). (c) Spheroid size (cells/well) (n = 6 compounds, 5 replicates per compound). (d) Clearing (n = 6 compounds, 9 replicates per compound). (e) Magnification (n = 6 compounds, 8 replicates per compound). (f) Representative

Hoechst and SYTO 14 images for the spheroid-size, clearing, and magnification conditions; scale bars as marked. (g) Median normalized intensity (I/I_0) versus imaging depth per stain at 5 µm (grey) and 1.5 µm (red) imaging *z*-steps; bands show the 95% bootstrap CI (n = 58 wells at 5 µm, n = 18 wells at 1.5 µm), shaded regions and asterisks mark depths where the two curves diverge significantly by a cluster-based permutation test. (h) Cells detected per well×slice versus imaging depth across clearing conditions (40/40%, 20/40%, None, 40/40% at 1.5 µm; n = 6 compounds, 5 replicates per compound); coloured bars mark depths where a curve diverges significantly from 40/40%. (* $p<0.05$, ** $p<0.01$, *** $p<0.001$). (g) Two representative examples of spheroids exposed to DMSO and imaged with an air (top) or a water immersion (bottom) objective, both at 20×. SYTO 14, Hoechst, and Phalloidin&WGA are shown. Scale bar is 50 µm and imaging depth is 30 µm. (h) Unlabeled (left) and labeled UMAPs (right) of scAgg morphological profiles. (i) Reproducibility of a single multi-well plate as measured by Spearman correlations. A correlation of 0 indicates spheroids are missing at that compound and concentration. Source data are provided as a Source Data file.

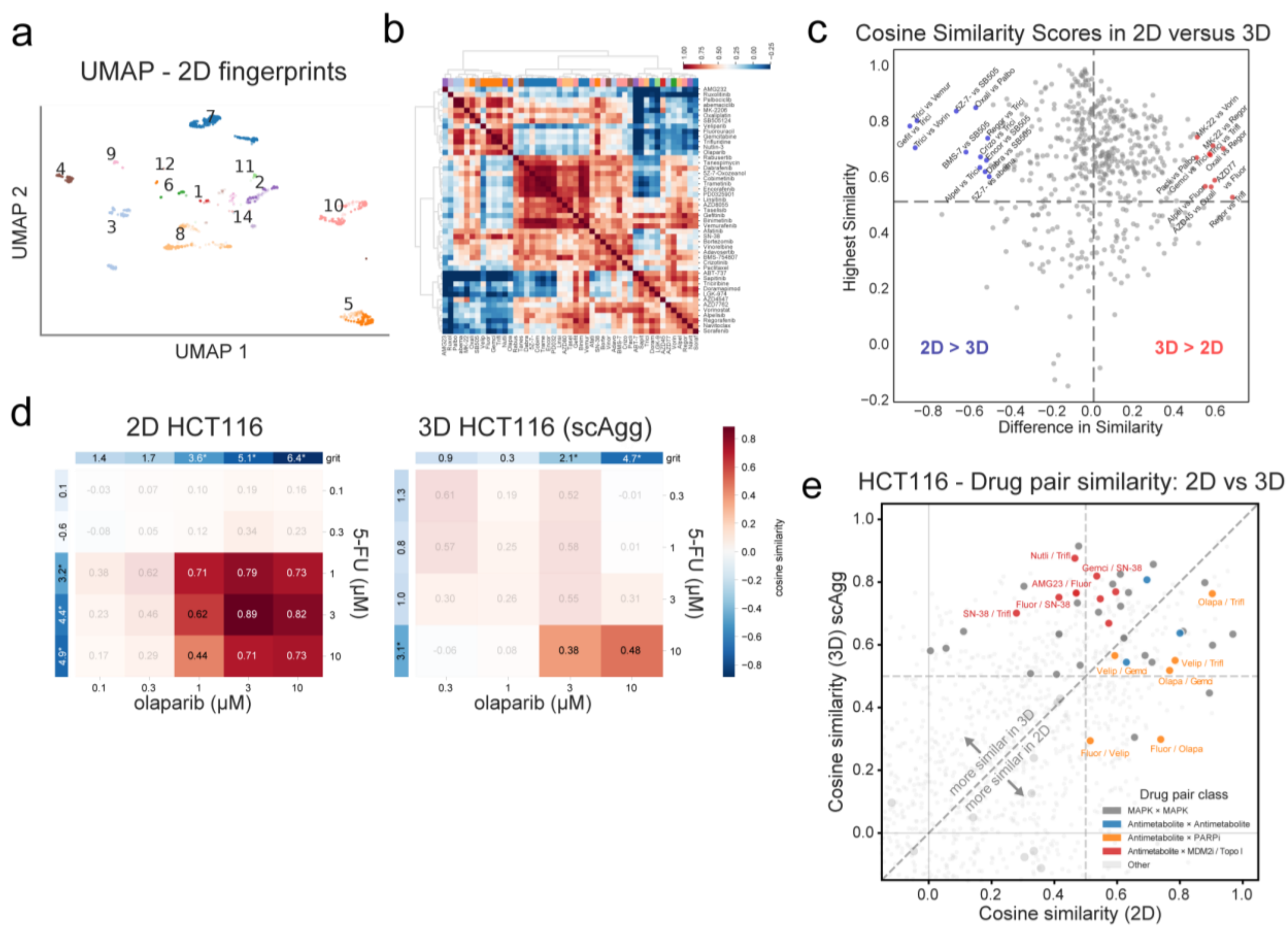

Supplementary Figure 5: Morphological profiles reveal different responses to DNA-damage-response compounds in spheroids versus adherent cells. (a) Semi-supervised UMAPs of 2D morphological profiles. (b) Heatmap displaying hierarchical clusters of cosine similarities for 2D profiles. (c) Comparison of cosine scores between pairs of compounds in 2D and 3D. The x-axis

shows the difference in similarity scores (3D – 2D), while the y-axis shows the highest similarity score across both modalities for each pair. We only included pairs with a cosine score of at least 0.5 in either 2D or 3D, and flagged as divergent those whose absolute between-modality difference (3D – 2D) fell in the top 5% (95th percentile) across all pairs. Panels A–C are based on HT29 cells. (d) Pairwise cosine similarity between 5-FU and olaparib across doses in HCT116, shown as heatmaps for 2D profiles (left) and single-cell aggregate (scAgg) 3D profiles (right); olaparib doses on the x-axis and 5-FU doses on the y-axis. The 2D-to-3D divergence is consistent across the dose range. (e) Cosine similarity for all compound pairs in HCT116, comparing 2D (x-axis) with scAgg 3D profiles (y-axis), color-coded by drug-pair class (MAPK inhibitor × MAPK inhibitor; antimetabolite × antimetabolite; antimetabolite × PARP inhibitor; antimetabolite × topoisomerase-I/MDM2 inhibitor). Throughout this figure, profiles are derived from n = 4 wells per condition in both 2D and 3D. Source data are provided as a Source Data file.

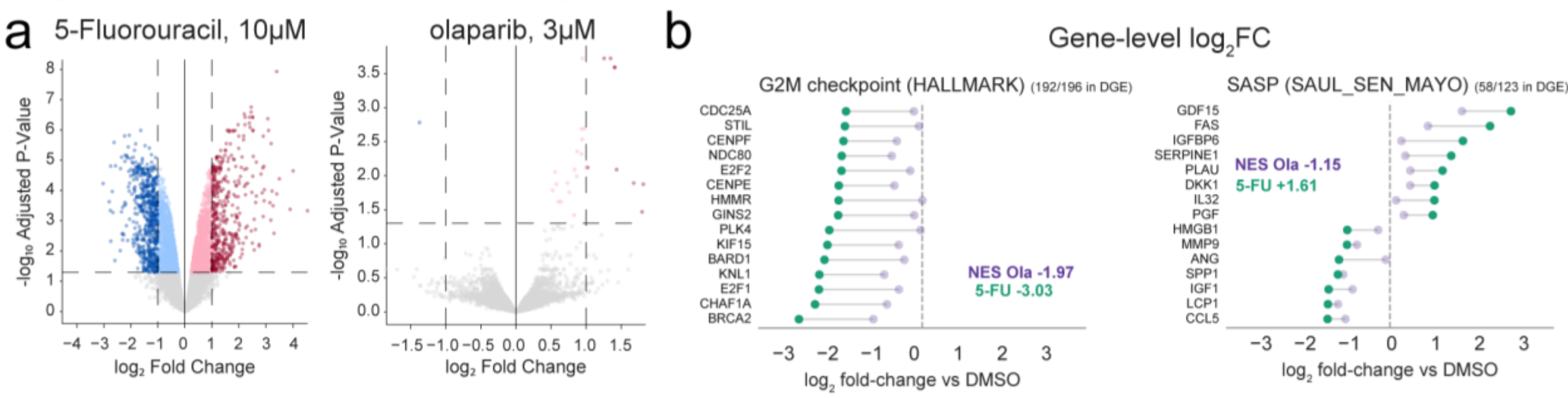

Supplementary Figure 6: Transcriptomic profiling supporting the 3D divergence between 5-FU and olaparib. (a) Volcano plots of differential gene expression for 5-FU (10 µM) and olaparib (3 µM), each versus DMSO control (*n* = 4 replicates per drug; *n* = 3 for DMSO, one replicate excluded). (b) Dot plot of Hallmark gene-set enrichment, highlighting the G2M checkpoint and senescence-associated secretory phenotype (SASP) signatures. Source data are provided as a Source Data file.

Supplementary Table 1: Second-site (AstraZeneca) cell seeding and compound treatment

Step	Details
Cells	300 HT29 cells in 30 µL medium per well
Plate / dispensing	384-well ultra-low attachment microplate (Corning 3830); Multidrop dispenser (Thermo Fisher Scientific)
Aggregation	Centrifuge 120 × g, 2 min; 24 h spheroid formation
Compounds	Berberine chloride, etoposide, fluphenazine, fenbendazole, nocodazole, sorbitol
Dose–response	10-point, 3-fold logarithmic dilutions from 10 µM top; each perturbation in triplicate
Compound dispensing	Echo 650 acoustic liquid handler (Labcyte, Beckman Coulter), 30 nL/well; final 0.1% DMSO
Incubation	37 °C, 5% CO_2, 48 h

Compound stocks	Prepared in DMSO by the AstraZeneca Compound Management department

Supplementary Table 2: Cell Painting assay parameters: 3D spheroid vs 2D monolayer, AstraZeneca

Step	3D spheroid	2D monolayer	AstraZeneca (second site)
Seeding	300 cells/well, ULA plate (Corning 3830); 24 h aggregation (120 × g, 2 min)	HCT116 2600 / HT29 2000 cells/well; PhenoPlate™ 384-well microplates (Revvity; 6057302)	300 HT29 cells/well in 30 µL, 384-well ULA (Corning 3830); 24 h aggregation (120 × g, 2 min)
MitoTracker	Included in the overnight stain mixture	Live stain: 40 µl in pre-warmed FluoroBrite, 20 min at 37 °C, before fixation	MitoTracker Deep Red (final 900 nM; Invitrogen M22426); 4× stock, 10 µL to medium; 1 h, 37 °C, before fixation
Wash / reagent volume	60 µl	80 µl	60 µl
Fixation	4% PFA, 1 h, RT	4% PFA, 20 min, RT	4% PFA (Histolab 02176), 1 h, RT
Permeabilization	0.2% Triton X-100, 1 h, RT	0.1% Triton X-100, 20 min, RT	0.2% Triton X-100 (Thermo Scientific 85111), 1 h, RT
Staining	20 µl stain mixture, 18 h, 4 °C	20 µl stain mixture, 20 min, RT	20 µl stain mixture (Suppl. Table 2 minus MitoTracker), 18 h, 4 °C
Clearing	70 µl fructose–glycerol solution, 72 h, 4 °C	None	70 µl clearing solution; sealed, 72 h, 4 °C

Supplementary Table 3: Feature extraction and preprocessing: scAgg, MIP, and 2D

Parameter	3D single cells (scAgg)	Max-intensity projection (MIP)	2D monolayer
Image-analysis software	CellProfiler 4.2.1 + Pycytominer	CellProfiler 4.2.1 + Pycytominer	CellProfiler 4.2.5 + Pycytominer (QC in 4.0.7)
Segmentation	Cellpose v2.0 cyto2, retrained (nuclei: Hoechst; cells: PHA+WGA)	Per-spheroid segmentation	Cellpose v2.0 cyto2, retrained per cell line
Illumination correction	None	None	Yes (Rietdijk et al., 2022)

Parameter	3D single cells (scAgg)	Max-intensity projection (MIP)	2D monolayer
Quality control	None	PowerLogLogSlope per channel; flag >93rd percentile, exclude if flagged >1×; blurry spheroids removed	Outlier-based flags based on QC pipeline
Features extracted	Shape, intensity, texture, granularity, radial distribution, colocalization, neighbors	Shape, intensity, texture	Shape, intensity, texture, granularity, radial distribution, colocalization, neighbors
Aggregation	Median per section → standardize → median per well	Per spheroid (z collapsed by projection)	Mean over sites, then median over wells
Standardization	Per plate and per optical section	Per plate	Per plate
Clipping	±40	±40	±40
Feature selection	Variance + correlation threshold; dropNA (Pycytominer)	Blocklist + variance + correlation threshold	Blocklist + variance + correlation threshold

Supplementary Table 4: The EdU and γH2AX assay. Staining, imaging, and analysis details

Step	Details
Cells & treatment	HCT116, 300 cells/well (Corning 3830 ULA), 24 h; then 48 h with 5-fluorouracil 10 µM, olaparib 3 µM, or etoposide 2.5 µM (0.1% DMSO; 4× cocktail, 10 µL/well, ViaFlo384)
EdU pulse	5 µM, 2 h, 37 °C, 5% CO_2
Fixation	4% PFA, 20 min, RT
Permeabilization / block	PBS + 0.2% Triton X-100 + 1% BSA, 1 h, RT
EdU detection	Click-iT EdU Alexa Fluor 647 HCS Assay (Thermo C10356, Components B–E), 1 h, RT, dark
γH2AX primary	anti-phospho-γH2AX (Ser139) rabbit mAb (CST #9718T, Lot 25), 1:500, 48 h, 4 °C
γH2AX secondary	goat anti-rabbit Alexa Fluor 488 (Invitrogen A-11034), 1:500, 2 h, RT, dark
Nuclei	Hoechst 33342, 1:1000, 1 h
Clearing	40% glycerol/fructose, equilibrated 2 d (as Cell Painting)
Imaging	Spinning-disk confocal, 30× (0.227 µm/px); HOECHST/γH2AX/EdU (Suppl. Table 2); 12-bit, 10 optical sections; HOECHST-channel spheroid detection (as Cell Painting)
Image analysis	Fiji rolling-ball background subtraction (radius 50 HOECHST/EdU, 5 γH2AX); CellProfiler 4.2.1 + Cellpose nuclear model; γH2AX spots via IdentifyPrimaryObjects (2–15 px; Robust Background 0.0004–1.0); EdU^+ by Otsu on log nuclear EdU; per-spheroid metrics aggregated over z
Statistics	n = 13 spheroids (one per well) per treatment, except etoposide (n = 8; remaining spheroids lost during processing). Kruskal–Wallis, then pairwise vs DMSO by Mann–Whitney U (Bonferroni) only where $p<0.05$
