# Supplementary material for: High-content morphological profiling by Cell Painting in 3D spheroids
Source: Nat Commun. 2026 Oct 21;17:10790. doi: 10.1038/s41467-026-78596-5 (PMC13686983; doi:10.1038/s41467-026-78596-5)
Supplement: Supplementary file 2 [file 41467_2026_78596_MOESM2_ESM.pdf]

Description of Additional Supplementary Files

Title: Supplementary Data 1

Description: Annotated compound library. The 52 screened small molecules, each with supplier and catalogue number, plate location, stock formulation, nominal target and pathway class (14 classes), reported mechanism and potency, highest clinical phase reached, and physicochemical and identifier data (molecular weight, formula, salt form, CAS number, DMSO and water solubility, SMILES, InChIKey, ALogP, hydrogen-bond counts, rotatable bonds, supplier URL).

Title: Supplementary Data 2

Description: Structured summary of the small-molecule screening data: Assay type and full protocol, biological system, measured features and the two preprocessing strategies, library composition and source, plate format, concentrations, controls, instrumentation and software, quality control, correction and normalization, the phenotypic activity criterion and downstream analyses.

Title: Supplementary Data 3

Description: Cell Painting staining reagents and image-acquisition settings (Excel, three sheets: 3D spheroids, 2D monolayers for HCT116 and HT29, and 3D at the second site). For each of the six imaged compartments: dye, catalogue number, solvent, final concentration, excitation wavelength or laser line, dichroic mirror, emission and excitation filters, exposure time and laser power. The 2D sheet also gives per-channel Z-offsets.
