# Supplementary material for: High-content morphological profiling by Cell Painting in 3D spheroids
Source: Nat Commun. 2026 Oct 21;17:10790. doi: 10.1038/s41467-026-78596-5 (PMC13686983; doi:10.1038/s41467-026-78596-5)
Supplement: Supplementary file 3 [file 41467_2026_78596_MOESM6_ESM.pdf]

nature portfolio

Corresponding author(s): Christa Ringers

Last updated by author(s): Aug 7, 2026

Reporting Summary

Nature Portfolio wishes to improve the reproducibility of the work that we publish. This form provides structure for consistency and transparency in reporting. For further information on Nature Portfolio policies, see our Editorial Policies and the Editorial Policy Checklist.

Statistics

For all statistical analyses, confirm that the following items are present in the figure legend, table legend, main text, or Methods section.

n/a	Confirmed	
☐	☒	The exact sample size (*n*) for each experimental group/condition, given as a discrete number and unit of measurement
☐	☒	A statement on whether measurements were taken from distinct samples or whether the same sample was measured repeatedly
☐	☒	The statistical test(s) used AND whether they are one- or two-sided<br>*Only common tests should be described solely by name; describe more complex techniques in the Methods section.*
☒	☐	A description of all covariates tested
☐	☒	A description of any assumptions or corrections, such as tests of normality and adjustment for multiple comparisons
☐	☒	A full description of the statistical parameters including central tendency (e.g. means) or other basic estimates (e.g. regression coefficient) AND variation (e.g. standard deviation) or associated estimates of uncertainty (e.g. confidence intervals)
☐	☒	For null hypothesis testing, the test statistic (e.g. *F*, *t*, *r*) with confidence intervals, effect sizes, degrees of freedom and *P* value noted<br>*Give P values as exact values whenever suitable.*
☒	☐	For Bayesian analysis, information on the choice of priors and Markov chain Monte Carlo settings
☐	☒	For hierarchical and complex designs, identification of the appropriate level for tests and full reporting of outcomes
☐	☒	Estimates of effect sizes (e.g. Cohen's *d*, Pearson's *r*), indicating how they were calculated

Our web collection on statistics for biologists contains articles on many of the points above.

Software and code

Policy information about availability of computer code

Data collection	Image acquisition and instrument control relied on commercial software. Spheroid detection and autofocus were implemented as a custom protocol in Nikon NIS-Elements with the JOBS and GA3 modules (v5.46.02), and Cell Painting plates were imaged on a Nikon ECLIPSE Ti2-E. Second-site acquisition used the Yokogawa CV8000 software. Compound and reagent dispensing used the I.DOT (Dispendix) and Echo 650 (Labcyte/Beckman Coulter) control software. Plate layouts were designed with PLAID.
Data analysis	mage segmentation and morphological feature extraction used the open-source tools Cellpose v2.0 (cyto2 model, retrained per cell line; nuclei model for the EdU/γH2AX assay) and CellProfiler (v4.2.1 for scAgg and MIP profiles, v4.2.5 for 2D, quality control in v4.0.7), with background subtraction performed in Fiji/ImageJ v1.54u2. Expert reference annotations were made in the Cellpose GUI. Isotropic resampling for orthogonal-slice display used Clij2. Single-cell profiles were aggregated and preprocessed with Pycytominer v0.2.0, and grit scores were computed with cytominer-eval.<br><br>Downstream analysis was carried out in Python 3.10 (Jupyter/JupyterLab v4.4.5) using numpy v1.22.0, scipy v1.7.3, scikit-learn, seaborn v0.13.2, and umap-learn v0.5.3. RNA-seq differential expression and gene-set enrichment used edgePython v0.2.5 and GSEApy v0.12 (against the MSigDB Hallmark gene set). Drug-target diagrams were generated with the clue.io web tool. Custom code is available at https://github.com/pharmbio/colopaint3D (analysis pipelines) and the SphereDetect autofocus and spheroid-detection routine at https://github.com/pharmbio/SphereDetect.

For manuscripts utilizing custom algorithms or software that are central to the research but not yet described in published literature, software must be made available to editors and reviewers. We strongly encourage code deposition in a community repository (e.g. GitHub). See the Nature Portfolio guidelines for submitting code & software for further information.

Data

Policy information about availability of data

All manuscripts must include a data availability statement. This statement should provide the following information, where applicable:

- Accession codes, unique identifiers, or web links for publicly available datasets
- A description of any restrictions on data availability
- For clinical datasets or third party data, please ensure that the statement adheres to our policy

The imaging data generated in this study have been deposited in the BioImage Archive database under accession code S-BIAD2254 [https://www.ebi.ac.uk/biostudies/bioimages/studies/S-BIAD2254], a repository previously described 113. The bulk RNA-seq data generated in this study have been deposited in the NCBI Gene Expression Omnibus database under accession code GSE343958 [https://www.ncbi.nlm.nih.gov/geo/query/acc.cgi?acc=GSE343958]. The BioImage Archive deposit comprises raw 16-bit OME-TIFF images, per-compartment single-cell CellProfiler feature tables (cells, cytoplasm, and nuclei), and the corresponding Cellpose segmentation masks, organised per acquisition plate; a separate detection demonstrator dataset is also included. Folder structure and file naming are documented in the deposit's README. The source data underlying Figures 2–6 and Supplementary Figures 1–6 are provided in the Source Data file. All data generated in this study are publicly available without restriction.

Research involving human participants, their data, or biological material

Policy information about studies with human participants or human data. See also policy information about sex, gender (identity/presentation), and sexual orientation and race, ethnicity and racism.

Reporting on sex and gender	sex was not considered as a variable in the study design because the profiling method is cell-line agnostic and not intended to test sex-dependent effects
Reporting on race, ethnicity, or other socially relevant groupings	NA
Population characteristics	NA
Recruitment	NA
Ethics oversight	NA

Note that full information on the approval of the study protocol must also be provided in the manuscript.

Field-specific reporting

Please select the one below that is the best fit for your research. If you are not sure, read the appropriate sections before making your selection.

[x] Life sciences [ ] Behavioural & social sciences [ ] Ecological, evolutionary & environmental sciences

For a reference copy of the document with all sections, see nature.com/documents/nr-reporting-summary-flat.pdf

Life sciences study design

All studies must disclose on these points even when the disclosure is negative.

Sample size	No statistical method was used to predetermine sample size. Sample sizes were chosen based on standard practice for high-content morphological profiling and the throughput of the 384-well screening format. Each well contained a single spheroid, from which Cellpose resolved on average 1,425 single-cell (2D segmentation-object) instances per spheroid in HCT116 and 854 in HT29; after correcting for the estimated 3–4× cross-section duplication, this corresponds to approximately 400 (HCT116) and 330 (HT29) unique cells per spheroid, providing several hundred single-cell measurements per well and thousands per condition — sufficient for robust population-level morphological aggregation. Each compound was screened in four replicate wells (two biological × two technical replicates) across 3 plates, with 20 interspersed DMSO control wells per plate. These numbers are consistent with established Cell Painting studies and were sufficient to resolve reproducible phenotypic clustering
Data exclusions	Data were excluded based on a standard QC pipeline, and based on phenotypic inactivity (grit < 1.96). Bulk RNA seq was excluded based on low read count.
Replication	Experiments were performed across 2 independent biological replicates and 2 technical/plate replicates. Attempts at replication were successful.
Randomization	Random allocation is not applicable to any experiment in this study: these are compound treatments of immortalized cell lines, not experiments on subjects. Within each experiment all wells derive from a common cell suspension per cell line, and there are no pre-existing subject characteristics to balance across groups. Cell line and compound are studied variables, not covariates. The relevant risk is positional bias, controlled at the design stage in each case.<br><br>Spheroid Cell Painting screen (Figures 2–5, 6e). Well assignments were generated with PLAID (Francisco Rodríguez et al., 2023) to distribute compounds, doses and controls across each plate; outer wells were excluded; each plate carried 20 interspersed DMSO controls; two biological replicates (independent thaws, separate plates) and two technical replicates per condition. Per-plate and per-section normalization

were applied during feature processing (Figure 2g).

2D adherent Cell Painting (Figure 5). Same design and layout approach, acquired as a separate run of four plates with the same replicate structure.

EdU/γH2AX (Figure 6a,b). Spheroids from a single common suspension were seeded across wells, conditions assigned by plate position, outer wells excluded; single run, single thaw.

Bulk RNA-seq (Figure 6c,d). Two independently thawed vials each seeded across two plates, 128 wells per condition per plate pooled before extraction; conditions assigned by plate position.

Blinding

Blinding was not relevant to this study. Image acquisition, segmentation, and feature extraction were performed by automated pipelines (NIS-Elements JOBS, Cellpose, CellProfiler) applied identically and without operator judgment to all conditions, so investigator bias in data collection or analysis was not a factor.

Reporting for specific materials, systems and methods

We require information from authors about some types of materials, experimental systems and methods used in many studies. Here, indicate whether each material, system or method listed is relevant to your study. If you are not sure if a list item applies to your research, read the appropriate section before selecting a response.

Materials & experimental systems

n/a	Involved in the study
☐	☒ Antibodies
☐	☒ Eukaryotic cell lines
☒	☐ Palaeontology and archaeology
☒	☐ Animals and other organisms
☒	☐ Clinical data
☒	☐ Dual use research of concern
☒	☐ Plants

Methods

n/a	Involved in the study
☒	☐ ChIP-seq
☒	☐ Flow cytometry
☒	☐ MRI-based neuroimaging

Antibodies

Antibodies used

nti-phospho-Histone H2A.X (Ser139), rabbit monoclonal, clone 20E3, Cell Signaling Technology cat. #9718T, lot 25, used at 1:500 (both primary and secondary)

Validation

The anti-phospho-γH2A.X (Ser139) antibody (CST #9718, clone 20E3) is a rabbit IgG monoclonal raised against a synthetic phosphopeptide corresponding to residues surrounding Ser139 of human H2A.X. The manufacturer states it detects endogenous H2A.X only when phosphorylated at Ser139, and validates it for western blot, immunohistochemistry (paraffin and frozen), immunocytochemistry/immunofluorescence and flow cytometry in human, mouse, rat and monkey. Phospho-specificity is supported on the manufacturer's datasheet by loss of signal following lambda-phosphatase treatment. The clone is widely cited in the DNA-damage literature. Manufacturer validation data: https://www.cellsignal.com/products/primary-antibodies/phospho-histone-h2a-x-ser139-20e3-rabbit-mab/9718

Validation in this study: the antibody was used for whole-mount immunofluorescence in cleared HCT116 spheroids. Specificity in this context is supported by the expected biological response. Etoposide, a topoisomerase II poison, produced a marked increase in γH2AX foci per nucleus relative to DMSO (Figure 6b), consistent with its known induction of double-strand breaks.

Eukaryotic cell lines

Policy information about cell lines and Sex and Gender in Research

Cell line source(s)

HT29 (ATCC HTB-38; Cellosaurus CVCL_0320): human colorectal adenocarcinoma cell line derived from a Caucasian female primary tumour. Sex of donor: female. HCT116 (ATCC CCL-247; Cellosaurus CVCL_0291): human colorectal carcinoma cell line derived from an adult male. Cellosaurus/ENCODE list the donor as a 48 year old male. Sex of donor: male.

Authentication

STR profiling

Mycoplasma contamination

Tested negative

Commonly misidentified lines
(See ICLAC register)

NA

Plants

Seed stocks

Report on the source of all seed stocks or other plant material used. If applicable, state the seed stock centre and catalogue number. If plant specimens were collected from the field, describe the collection location, date and sampling procedures.

Novel plant genotypes

Describe the methods by which all novel plant genotypes were produced. This includes those generated by transgenic approaches, gene editing, chemical/radiation-based mutagenesis and hybridization. For transgenic lines, describe the transformation method, the number of independent lines analyzed and the generation upon which experiments were performed. For gene-edited lines, describe the editor used, the endogenous sequence targeted for editing, the targeting guide RNA sequence (if applicable) and how the editor was applied.

Authentication

Describe any authentication procedures for each seed stock used or novel genotype generated. Describe any experiments used to assess the effect of a mutation and, where applicable, how potential secondary effects (e.g. second site T-DNA insertions, mosiacism, off-target gene editing) were examined.
