# Supplementary material for: High-content morphological profiling by Cell Painting in 3D spheroids
Source: Nat Commun. 2026 Oct 21;17:10790. doi: 10.1038/s41467-026-78596-5 (PMC13686983; doi:10.1038/s41467-026-78596-5)
Supplement: Supplementary file 4 [file 41467_2026_78596_MOESM7_ESM.pdf]

nature portfolio

Peer Review File

High-content morphological profiling by Cell Painting in 3D spheroids.

Corresponding Author: Dr Christa Ringers

This file contains all reviewer reports in order by version, followed by all author rebuttals in order by version.

Version 0:

Reviewer comments:

Reviewer #1

(Remarks to the Author)
Ringers et al. present a workflow for applying the widely used CellPainting assay in 3D. This report is highly anticipated by the community, as several laboratories are seeking methods for morphological profiling in complex 3D cell culture assays. The CellPainting assay has been extensively used in 2D for various applications, including discovering compound modes of action and evaluating safety. Extending this protocol to 3D cell culture is particularly valuable, as more laboratories are adopting intricate in vitro models grown in 3D.

The authors implemented several modifications to the protocol. They extended the incubation times for the reagents, used plastic-compatible solutions for clearing, and developed a normalization strategy to compensate for signal degradation across the z-stack. Additionally, they employed a home-grown algorithm utilizing the FocusScore to optimize the acquisition of in-focus images of spheroids. Overall, their strategy is sound and has yielded valuable imaging data.

Building upon their developed protocol, the authors conducted a screen of 52 clinically approved compounds in both 2D and 3D in HT116 and HT29 cells. They investigated the differences between analyzing maximal projections of 3D stacks and single-cell 2D segmentation aggregation per organoid data sets. Furthermore, they compared the 2D dataset with the 2D aggregation dataset of spheroids to explore the variations in biological responses between 2D and 3D.

In summary, this manuscript is well-written and addresses a significant knowledge gap in the field of morphological profiling. The methodology is meticulously described, and the tools employed are at the forefront of morphological profiling. While I have only a few minor suggestions for recommending publication, I believe this work contributes significantly to the advancement of morphological profiling in 3D cell culture.

- It’s commendable that the authors implemented their workflow on a different system in a different location. However, the reporting of this experiment is quite brief and doesn’t provide enough details for readers to fully comprehend the implementation. I suggest either mentioning the implementation in the discussion section or adding more supplementary materials to enable reproducing the workflow.

- I was slightly confused about the flow of the manuscript when the 2D experiment was introduced. Initially, I thought it might be a copy-paste error until I realized it was a new dataset. The authors might consider introducing both datasets and the three analytical pipelines at the beginning to provide a clearer context for readers.
- The authors opted for 2D segmentation instead of 3D segmentation. Additionally, the z-stack step of 5µm is insufficient for achieving isotropic resolution. It would be beneficial to address the challenges posed by computational power for 3D analysis and the data storage requirements, including I/O, for large 3D isotropic datasets. These factors are crucial when evaluating the implementation of 3D biology.

(Remarks on code availability)
I have not reviewed the code, but most software used are publicly available and I have worked with them. The only home grown software is the autofocus routine, which I cannot test.

Reviewer #2

(Remarks to the Author)
The manuscript by Ringers et al describes an adaptation of the Cell Painting assay to 3D spheroids.
To achieve this, the authors present modifications on three levels: staining protocol, image acquisition and image analysis.
1) The authors present an optimized staining protocol (using longer incubation times with the dyes) which also integrates an existing tissue clearing method to achieve higher resolution of single cells upon imaging by confocal spinning disk microscopy.
2) The authors optimized automated image acquisition using a step-wise on the fly detection of organoids in the 3 D space. Their algorithm differs from existing methods by using image focus score as a guiding parameter as compared to using fluorescence intensity. However, the authors do not relate the performance of their method to the existing ones.
3) The authors then used all 13 confocal slices in the image analysis to enable the computing of single cell features. Cleverly, the authors realized that it is necessary to normalize the resulting morphological profiles to controls of the same slice to counteract the degrading image quality across slices.
The authors then benchmarked their single-cell derived morphological profiles against profiles derived from maximum intensity projections into 2 D which can be considered current state-of-art. Both approaches yielded very comparable results in their power of identifying compounds that induce a significant phenotypic change. However, the single-cell algorithm delivered morphological profiles with superior resolution as judged by a reproducibility comparison. This then translated favorably into the ability to detect pathway-specific phenotypic changes in a hierarchical clustering approach after introduction of class labels. However, this is not apparent in a dimension reduction approach.
The authors also show that their protocol is transferable to another lab.
Lastly, the authors reveal differential cellular responses upon treatment with DNA-damaging agents when comparing 3D versus 2D culture conditions. While it is well known that drug responses differ in 3D versus 2D, this has not been shown in a morphological profiling approach.
In conclusion the authors combine some valuable technical advancements to enable Cell Painting in organoids with analysis on the single cell level. However, as these technical details are either gradual increases or are derived from related fields, it can be questioned if this really substantiates a new framework that warrants publication in Nat.Comm.

Further minor issues:
• Details of the assay description are unclear. How many technical replicates were used? How many DMSO wells were used.
• l. 598 No Mitotracker staining is mentioned.
• Supplementary tables are missing. It is not possible to evaluate the explored 52 compounds: “how many are clinically relevant drugs”, which drugs are these and which are the preclinical compounds? The authors should add the assignment of these drugs to the respective pathways used for the clustering
• For the UMAP plots in Fig. 4: it would be very helpful know which drugs were assigned to which pathway.
• l.264 What means 1607 wells? per week? In the total study?
• Figure 3: incorrect labelling in the caption (C2=D?)
• The authors do not refer to some Supplementary data, for example Supplementary Figure 3B. Supplementary Figure 1 is missing or the numbering has to be adjusted.
• Does the difference in profile simiality for 5-Fu an olaparib in 2D and 3D is seen for other nucleosides vs. PARP inhibitors?

(Remarks on code availability)

Reviewer #3

(Remarks to the Author)
Ringers and colleagues present a scalable method for implementing Cell Paint on 3D spheroids. They present a high-throughput and scalable method to automatically detect, image and analyze different colorectal cancer cell lines grown in 2D and as 3D spheroids. The manuscript revolves around developing automated single cell analysis in 3D using Cell Profiler. The content of the manuscript connects neatly with the PIs interest, by utilizing AI and automation to improve in the end autonomous phenomics.
Overall, the strength of the manuscript is building an open-source automated pipeline for high-throughput 3D single cell phenomics that builds on the original Cell Paint protocol. There are however some criticisms in the strategy chosen by the authors. In particular, the current cell analysis set-up represents incremental advance over the original cell paint protocol, as the image analysis basically represents 2D cell paint from images taken from 3D structures.
Implementing the suggestions as outlined below, will improve the impact of the manuscript and elevate it from its current incremental advance over the classical 2D cell paint and align its strengths with the unique assets of popular 3D models.

Major comments:
1. The findings in the current manuscript focuses on homogenous 3D spheroid models, however using organoids as a 3D model would greatly increase the strength of the story. Foremost I would like to see that the Cell Paint is combined with antibody stainings (against cell type markers) to generate different cell profiles for different cell identities. Expanding this pipeline would maximize the potential to study cell biology in more advanced 3D structures, not only drug responses in cancer organoids but also to facilitate therapeutic therapies for diseases in ‘normal’ (non-transformed) tissues. To integrate antibody stainings, the authors can perhaps continue on the recent Cell Paint Plus application (von Coburg et al., Nat comms 2025). Combining cell phenotyping with for example, lineage markers, differentiation states, cell cycle status or

transcriptomic profiles would add a critical layer of biological interpretation. This is especially relevant when assessing drug responses, as different cell types within a spheroid or organoid may respond differently (persister cells). Including cell identity could help disentangle compound-specific effects from cell-type-specific responses.

2. Tissue clearing is brought as an essential part of the pipeline. However, since data is pooled one could scan more spheroids to reach the same conclusions. In particular since the imaging pipeline is fully automated, image acquisition could be easily expanded. Moreover, the authors even mention that water immersion already reaches higher mean FocusScore, questioning the need for the lengthy tissue clearing step. To evaluate the needs versus nice to haves, it is essential that investigations are performed into variables like impact of tissue clearing versus no clearing, restricting image acquisition to lower 'half' of the spheroids/organoids (or predefined depths like 30um into a spheroid) and compensating with scanning more objects, heterogeneity of cellular phenoscopes within and between independent spheroids/organoids (thus ideal number of cells analyzed per spheroid and number of spheroids) as well as the impact of relative positioning of cells within objects, and spheroid/organoid sizes (both in terms of biology as well as acquisition quality). Important is to define how do these variables combine for optimal data acquisition in terms of quality and quantity?

3. From a technical perspective, I understand that Cell Paint (cellpose + cell profiler) is applied on 2D excerpts (z-sections) that are taken from 3D spheroids. Can the authors generate a demonstrator dataset where the current pipeline (Cell paint on z-sections per 5um step size) is combined and compared with high resolution image analysis (40x) where cellular properties (cell profiler) are assigned to 3D rendered cell volumes (Nyquist step size acquisition)?

4. For a general reader, it will be informative to showcase how the dyes exactly stain individual cells in spheroids. High resolution images with 63x objectives would be informative. Also, what is the impact of image resolution on the ultimate phenoscaping outcome?

Minor comments:

Fig 2A-C don't add much to the main part of the manuscript, I would combine these in a single panel. Furthermore, adding high-resolution images of the individual dyes would add to clarity.

Fig 3A2 and B2: The bar graphs are hard to interpret, adding a threshold line for the grit score (> 1.96) can help with this.

Fig 4A-B1: The UMAPs are nice, however the colors are hard to connect to the legends. Adding a number in combination with the colors would help.

Fig 5 is nice to show the principle, however I believe it does not add much to the main rationale. Therefore, I would consider moving it to the supplement.

(Remarks on code availability)

Reviewer #4

(Remarks to the Author)
Comments for the Author

This manuscript extends Cell Painting to 3D spheroids by combining clearing, confocal optical sectioning, and automated single-cell analysis. The topic is timely and relevant for phenomics and preclinical screening. To further meet the journal's standards for rigor and reproducibility, several conclusions and figures would benefit from additional methodological detail and quantitative support.

Major Comments

The study currently presents a qualitative segmentation example (e.g., Fig. 2E). To enhance robustness, consider adding a small expert-annotated set and reporting quantitative metrics across depths and channels (e.g., IoU/Dice/F1), together with a discussion of how segmentation error propagates to features and clustering.

The wording "cells per spheroid" could be misinterpreted; it appears closer to a sum of per-section 2D segmentation instances, where the same cell may be counted across multiple sections. It may be clearer to phrase this as "2D segmentation object instances per spheroid," explicitly noting that this is not a unique 3D cell count; if feasible, a simple cross-section de-duplication estimate would be helpful.

Per-section normalization assumes similar technical attenuation across wells at the same depth. When compounds markedly alter spheroid optics (e.g., strong necrosis or vacuolization affecting scattering/refractive index), this assumption may introduce bias. It would help to acknowledge this as a methodological limitation and include a few robustness checks.

Fig. 2H. This figure is used to indicate a reduction of depth artefacts, but it is difficult to interpret rigorously in its current form. To make it more informative, please: (i) label axes with the percentage of variance explained by PC1/PC2 for both panels; (ii) clarify in the legend the granularity of each point (e.g., well-level aggregate vs per-section profile); and (iii) state whether

the two panels use a shared PCA loading basis or independent PCAs (if independent, note that axes are not directly comparable). If appropriate, learning a shared basis on combined data and reporting PC–section correlations or a simple variance-partition analysis would strengthen the claim.

In Fig. 4A1/B1, UMAP embeddings incorporate pathway labels at the embedding stage, which is helpful for visualization. To support statements about discovering pathway-level structure, it would be valuable to include fully unsupervised embeddings and quantify label enrichment (e.g., ARI/NMI, permutation tests). For reproducibility, please disclose key UMAP hyperparameters (n_neighbors, min_dist, metric, random_state) and provide the label file used for semi-supervision.

For the comparison between Fig. 4A2 and 4B2, the characterization of the MIP heatmap as a “single cluster” feels somewhat subjective. It may be clearer to specify clustering rules (distance, linkage, tree-cut height or dynamic tree cut), report the resulting number of clusters and separation metrics (e.g., silhouette score), and—under the same sample filter (e.g., grit $\geq$ 1.96)—use pathway labels as external truth for both MIP and scAgg.

In Fig. 6A, the text could be read as implying that lower grit in 3D directly reflects weaker biological responses. Since grit reflects both effect size and measurement noise, and 3D assays inherently face added noise (penetration, scattering, depth attenuation, segmentation uncertainty), it would strengthen the analysis to elaborate on these technical factors and, where possible, separate them from biology.

The differences shown in Fig. 6D–F are consistent with spatial proliferation and microenvironmental heterogeneity in spheroids. If the intent is to emphasize new mechanistic insight, some softening of language may help, alongside additional support such as depth-resolved proliferation readouts (e.g., EdU/Ki-67) or ring/zone morphology analyses to bridge morphology and mechanism.

To improve reproducibility, please provide and clearly link in the data availability/methods the following materials already referenced in the main text but not currently included with the submission: Supplementary Table 1 (compound list and doses) and Supplementary Table 2 (dyes/channels/filters/exposure settings).

Minor Comments

Clarify inclusion/exclusion criteria across analyses (e.g., whether grit < 1.96 is uniformly excluded), so readers can compare figures consistently.

Maintain consistent terminology (e.g., choose between “spheroid-wide MIP” and “MIP-based profiles” and use it throughout).

Improve figure legibility by adding scale bars, channel labels, and contrast adjustments, and indicate magnification and depth for all panels.

A few spelling/formatting items could be polished (e.g., “single-cel”).

For code usability and reuse, please add a small reproducibility bundle: (i) an environment lock file (requirements.txt/environment.yml), (ii) a tiny public test dataset with a clear README (install, data paths, one end-to-end example), and (iii) a short script/notebook that regenerates a small subset of panels. If feasible, expose core steps as simple documented functions and include one or two sanity checks.

I hope these suggestions help strengthen the manuscript, and I look forward to seeing a revised version.

(Remarks on code availability)
At present, the repository functions more as analysis scripts than a turnkey reproduction, and I did not attempt a full end-to-end run. A brief README covering installation and data paths, an environment lock file (requirements.txt or environment.yml), and a very small public test dataset would make it easier to try the code end-to-end. If feasible, a short script or notebook that regenerates a small subset of panels from the released feature tables would further lower the barrier to reuse.

Version 1:

Reviewer comments:

Reviewer #1

(Remarks to the Author)
The revised manuscript has been extended to include further validations of the original findings. This reinforces my earlier recommendation for publication and I have no further comments.

(Remarks on code availability)

Reviewer #2

(Remarks to the Author)
I would like to congratulate the authors on the successful improvement of their manuscript.
All raised issues have been satisfyingly addressed. The functional validation of the divergent profiles of the DNA-damage-response agents 5-Fluorouracil and Olaparib in 3D is impressive.
Publication in Nat Comm is warranted.

(Remarks on code availability)

Reviewer #3

(Remarks to the Author)
I thank the authors for their revisions and for the thorough and thoughtful responses to my comments and suggestions. I appreciate the explanation why the authors deliberately chose spheroids as their model system for developing an accessible and easily scalable high-throughput assay. At the same time, it is unfortunate that most of the suggestions to gain impact (like exploring applications on organoids, cell types etc) were textually argued to be beyond the scope of the paper. That may be true, but the current focus on spheroids significantly downplays the impact of the manuscript as the technical gains are more like refinements than a substantial novel methodology.
Second, a significant downfall of the manuscript is the absence of a direct comparison of their approach with a true volumetric 3D segmentation and downstream analysis strategy. While the authors clearly explain that volumetric imaging has significant disadvantages in terms of image acquisition and assay practicality, it now remains handwaving how much superior performance a true volumetric 3D analysis pipeline could achieve versus its costs in terms of big data, compute and practicality. A head-to-head comparison could have been very instrumental in guiding future researchers to select their method of choice.
For instance, demonstrating that the presented method achieves (near)-equivalent outcomes to those obtained through true volumetric 3D segmentation, while being far superior in terms of practicality and scaling would substantially strengthen the impact of the paper and broader applicability and adoptability of their approach.

(Remarks on code availability)

Reviewer #4

(Remarks to the Author)
Thank you for the careful revision. Most of my previous concerns have been addressed. I only have a few remaining points.

1. The depth-normalization analysis is clearer, but I am not sure whether the same DMSO controls were used both to calculate and evaluate the correction. If so, the complete loss of the depth trend would be partly expected. Please clarify this point and consider changing “remove” to “mitigate” if no independent validation was used.
2. Please clarify the hierarchical clustering method. The manuscript mentions Ward linkage together with cosine similarity, which is not a standard combination. A brief clarification, or a robustness check using a linkage method compatible with cosine distance, would be helpful.
3. The new EdU, γH2AX, and RNA-seq data strengthen the biological interpretation. However, “p53-dependent” seems somewhat strong because HCT116 and HT29 are not isogenic. Please soften this wording.
4. The supplementary compound table is now included, but I could not find the exact four concentrations used for each compound in the main 2D and 3D screens. Please add these values or indicate clearly where they are listed.

(Remarks on code availability)

Open Access This Peer Review File is licensed under a Creative Commons Attribution 4.0 International License, which permits use, sharing, adaptation, distribution and reproduction in any medium or format, as long as you give appropriate credit to the original author(s) and the source, provide a link to the Creative Commons license, and indicate if changes were made.
In cases where reviewers are anonymous, credit should be given to 'Anonymous Referee' and the source.
The images or other third party material in this Peer Review File are included in the article's Creative Commons license, unless indicated otherwise in a credit line to the material. If material is not included in the article's Creative Commons license and your intended use is not permitted by statutory regulation or exceeds the permitted use, you will need to obtain permission directly from the copyright holder.
To view a copy of this license, visit https://creativecommons.org/licenses/by/4.0/

We thank the four reviewers for their thorough and constructive evaluation of our manuscript, and for recognizing the value of the developed protocols. We are grateful for the suggestions, which have helped us strengthen the work.

In response, we did two main things. First, we turned the central 2D-versus-3D divergence into a mechanistic result: 5-Fluorouracil and Olaparib, near-indistinguishable in 2D, diverge in 3D, and orthogonal EdU, γH2AX, and RNA-seq (new Fig. 6) trace this to a p53-dependent difference: 5-Fluorouracil drives a stronger p53/p21 response despite less DNA damage. This turns a phenotypic observation into a literature-grounded hypothesis no 2D or projection profile could resolve. Second, we mapped where the method works and where it does not (clearing requirements, acquisition trade-offs, resolution limits), backed by an expert-annotated segmentation benchmark (r = 0.93–0.99). Together these sharpen the core contribution: single-cell resolution inside intact 3D spheroids at screening scale (six 384-well plates) on standard spinning-disk hardware most labs own, independently validated by Nikon and now a standard option. Light-sheet platforms reach higher resolution but not this throughput, so the approaches are complementary, not competing.

Our point-by-point responses to each comment are provided below. Reviewer comments are shown; our responses follow in blue text. All corresponding changes in the manuscript are marked with tracked changes.

REVIEWER COMMENTS

Reviewer #1 (Remarks to the Author):

Ringers et al. present a workflow for applying the widely used CellPainting assay in 3D. This report is highly anticipated by the community, as several laboratories are seeking methods for morphological profiling in complex 3D cell culture assays. The CellPainting assay has been extensively used in 2D for various applications, including discovering compound modes of action and evaluating safety. Extending this protocol to 3D cell culture is particularly valuable, as more laboratories are adopting intricate in vitro models grown in 3D.

The authors implemented several modifications to the protocol. They extended the incubation times for the reagents, used plastic-compatible solutions for clearing, and developed a normalization strategy to compensate for signal degradation across the z-stack. Additionally, they employed a home-grown algorithm utilizing the FocusScore to optimize the acquisition of in-focus images of spheroids. Overall, their strategy is sound and has yielded valuable imaging data.

Building upon their developed protocol, the authors conducted a screen of 52 clinically approved compounds in both 2D and 3D in HT116 and HT29 cells. They investigated the differences between analyzing maximal projections of 3D stacks and single-cell 2D segmentation aggregation per organoid data sets. Furthermore, they compared the 2D dataset with the 2D aggregation dataset of spheroids to explore the variations in biological responses between 2D and 3D.

In summary, this manuscript is well-written and addresses a significant knowledge gap in the field of morphological profiling. The methodology is meticulously described, and the tools employed are at the forefront of morphological profiling. While I have only a few minor suggestions for recommending publication, I believe this work contributes significantly to the advancement of morphological profiling in 3D cell culture.

- It’s commendable that the authors implemented their workflow on a different system in a different location. However, the reporting of this experiment is quite brief and doesn’t provide enough details for readers to fully comprehend the implementation. I suggest either mentioning the implementation in the discussion section or adding more supplementary materials to enable reproducing the workflow.

We have moved the figure to the supplementary per recommendation of reviewer 3. In addition, we have expanded on the methods for the workflow implementation – which were also moved to the supplementary.

- I was slightly confused about the flow of the manuscript when the 2D experiment was introduced. Initially, I thought it might be a copy-paste error until I realized it was a new dataset. The authors might consider introducing both datasets and the three analytical pipelines at the beginning to provide a clearer context for readers.

We added an overview of both datasets and analytical pipelines to Figure 1 and its description. In addition, we signposted the existence of the 2D dataset earlier in the manuscript (l. 270) and included a bridging sentence (l. 430).

- The authors opted for 2D segmentation instead of 3D segmentation. Additionally, the z-stack step of 5µm is insufficient for achieving isotropic resolution. It would be beneficial to address the challenges posed by computational power for 3D analysis and the data storage requirements, including I/O, for large 3D isotropic datasets. These factors are crucial when evaluating the implementation of 3D biology.

This is a fair point. We have added a short paragraph to the discussion to highlight this challenge (l. 602).

Reviewer #1 (Remarks on code availability):

I have not reviewed the code, but most software used are publicly available and I have worked with them. The only home grown software is the autofocus routine, which I cannot test.

We have uploaded an example detection dataset to the BioImage Archive (10.6019/S-BIAD2254), and updated the SphereDetect code (https://github.com/pharmbio/SphereDetect) with this example.

Reviewer #2 (Remarks to the Author):

The manuscript by Ringers et al describes an adaptation of the Cell Painting assay to 3D spheroids.
To achieve this, the authors present modifications on three levels: staining protocol, image acquisition and image analysis.
1) The authors present an optimized staining protocol (using longer incubation times with the dyes) which also integrates an existing tissue clearing method to achieve

higher resolution of single cells upon imaging by confocal spinning disk microscopy.
2) The authors optimized automated image acquisition using a step-wise on the fly detection of organoids in the 3 D space. Their algorithm differs from existing methods by using image focus score as a guiding parameter as compared to using fluorescence intensity. However, the authors do not relate the performance of their method to the existing ones.

To our knowledge, existing spheroid detection algorithms are not publicly available. We have therefore uploaded an example dataset to enable benchmarking by future researchers. The method has also been independently tested by Nikon, who found it robust and are integrating it as a standard option in their system; an application note describing this is in preparation.

3) The authors then used all 13 confocal slices in the image analysis to enable the computing of single cell features. Cleverly, the authors realized that it is necessary to normalize the resulting morphological profiles to controls of the same slice to counteract the degrading image quality across slices.
The authors then benchmarked their single-cell derived morphological profiles against profiles derived from maximum intensity projections into 2 D which can be considered current state-of-art. Both approaches yielded very comparable results in their power of identifying compounds that induce a significant phenotypic change. However, the single-cell algorithm delivered morphological profiles with superior resolution as judged by a reproducibility comparison. This then translated favorably into the ability to detect pathway-specific phenotypic changes in a hierarchical clustering approach after introduction of class labels. However, this is not apparent in a dimension reduction approach.

We thank the reviewer for this observation. To address the reviewer’s concern, we added an unsupervised UMAP (Figure 4A2-4B2), in which the pathway-specific phenotypic changes are now more apparent.

The authors also show that their protocol is transferable to another lab.
Lastly, the authors reveal differential cellular responses upon treatment with DNA-damaging agents when comparing 3D versus 2D culture conditions. While it is well known that drug responses differ in 3D versus 2D, this has not been shown in a morphological profiling approach.
In conclusion the authors combine some valuable technical advancements to enable Cell Painting in organoids with analysis on the single cell level. However, as these technical details are either gradual increases or are derived from related fields, it can be questioned if this really substantiates a new framework that warrants publication in Nat.Comm.

We thank the reviewer for recognizing the transferability and the novelty of demonstrating differential 2D-versus-3D drug responses by morphological profiling.

To our knowledge this is the first scalable single-cell Cell Painting in 3D spheroids on a high-content spinning-disk confocal, and it surfaced biology that neither 2D nor projections could resolve: 5-Fluorouracil and Olaparib are near-indistinguishable in 2D and fall below threshold in intensity-projections (grit 1.05 and 0.98), yet diverge in 3D. In the revised manuscript we substantiate this divergence with orthogonal EdU and γH2AX immunostaining and bulk RNA-seq (Fig. 6), which confirm it is genuine rather than an imaging artifact and let us move from a phenotypic observation to a narrowed, literature-grounded hypothesis: a p53-dependent processing difference (lost in p53-mutant HT29) in which 5-Fluorouracil drives the stronger p53/p21 response despite less DNA damage than Olaparib. We achieve this while recovering single-cell resolution on a spinning-disk confocal most Cell Painting labs already own, and remaining compatible with standard Cell Painting.

Several of these observations have precedent individually but have not been brought together in one 3D model and framework. Given that why 5-Fluorouracil works remains incompletely understood, a method that surfaces format-dependent inconsistencies in established agents offers a useful entry point for mechanistic follow-up.

Further minor issues:

- Details of the assay description are unclear. How many technical replicates were used? How many DMSO wells were used. We used 2 biological and 2 technical replicates per condition, and included twenty wells of DMSO for each plate. This information is now iterated in the main text and the Methods for clarity. (l. 273 & l. 677)
- l. 598 No Mitotracker staining is mentioned. We have adjusted the Methods for clarity.
- Supplementary tables are missing. It is not possible to evaluate the explored 52 compounds: “how many are clinically relevant drugs”, which drugs are these and which are the preclinical compounds? The authors should add the assignment of these drugs to the respective pathways used for the clustering We ensured that the supplementary material is now properly attached. We sourced the clinical status from the Drug Repurposing Hub, which we now indicated in the text. Among the compounds, there are 27 launched drugs, 19 in clinical trials, and 6 preclinical drugs.
- For the UMAP plots in Fig. 4: it would be very helpful know which drugs were assigned to which pathway. We have added the supplementary information. You will find the pathway assignment in the Supplementary Table 1, under the Pathway column.
- l.264 What means 1607 wells? per week? In the total study? These wells refer to the profiling experiment in l. 280. We clarified the text now to mention the more conventional six 384-well plates.
- Figure 3: incorrect labelling in the caption (C2=D?) We thank the reviewer for catching

this mistake. We have corrected it.
• The authors do not refer to some Supplementary data, for example Supplementary Figure 3B. Supplementary Figure 1 is missing or the numbering has to be adjusted.
We have now referred to Supplementary Figure 1.
• Does the difference in profile similarity for 5-Fu an Olaparib in 2D and 3D is seen for other nucleosides vs. PARP inhibitors?
We thank the reviewer for this question. We plotted drug pairs by similarity in 2D and 3D, and color-coded them based on their class, including antimetabolite × PARP inhibitor, in Supplementary Figure 5D. The 2D-to-3D divergence follows a more general pattern. In 2D, antimetabolites (5-Fluorouracil, Gemcitabine, and Trifluridine) and PARP inhibitors (Olaparib, Veliparib) converge. In 3D, antimetabolites and MDM2/Top1 inhibitors converge.

Reviewer #3 (Remarks to the Author):

Ringers and colleagues present a scalable method for implementing Cell Paint on 3D spheroids. They present a high-throughput and scalable method to automatically detect, image and analyze different colorectal cancer cell lines grown in 2D and as 3D spheroids. The manuscript revolves around developing automated single cell analysis in 3D using Cell Profiler. The content of the manuscript connects neatly with the PIs interest, by utilizing AI and automation to improve in the end autonomous phenomics. Overall, the strength of the manuscript is building an open-source automated pipeline for high-throughput 3D single cell phenomics that builds on the original Cell Paint protocol. There are however some criticisms in the strategy chosen by the authors. In particular, the current cell analysis set-up represents incremental advance over the original cell paint protocol, as the image analysis basically represents 2D cell paint from images taken from 3D structures.
Implementing the suggestions as outlined below, will improve the impact of the manuscript and elevate it from its current incremental advance over the classical 2D cell paint and align its strengths with the unique assets of popular 3D models.

Major comments:

1. The findings in the current manuscript focuses on homogenous 3D spheroid models, however using organoids as a 3D model would greatly increase the strength of the story. Foremost I would like to see that the Cell Paint is combined with antibody stainings (against cell type markers) to generate different cell profiles for different cell identities. Expanding this pipeline would maximize the potential to study cell biology in more advanced 3D structures, not only drug responses in cancer organoids but also to facilitate therapeutic therapies for diseases in ‘normal’ (non-transformed) tissues. To

integrate antibody stainings, the authors can perhaps continue on the recent Cell Paint Plus application (von Coburg et al., Nat comms 2025). Combining cell phenotyping with for example, lineage markers, differentiation states, cell cycle status or transcriptomic profiles would add a critical layer of biological interpretation. This is especially relevant when assessing drug responses, as different cell types within a spheroid or organoid may respond differently (persister cells). Including cell identity could help disentangle compound-specific effects from cell-type-specific responses.

We thank the reviewer for these suggestions, which prompted us to probe the biology further and to clarify why a homogeneous spheroid was a deliberate choice for this method-development study.

Added biological interpretation. To add depth to the 5-Fluorouracil/Olaparib divergence, we incorporated orthogonal readouts. EdU and γH2AX staining showed Olaparib produced more DNA-damage foci (4.8 vs. 1.9 γH2AX foci/nucleus; $p < 0.001$), while 5-Fluorouracil imposed the stronger S-phase entry loss (7.7% vs. 16.3% EdU^{+}; $p < 0.001$; Fig. 6A–B). Bulk RNA-seq corroborated a p53/p21 program engaged more strongly by 5-Fluorouracil and dependent on p53 status, lost in p53-mutant HT29 (Fig. 6C–E). This moves the result from a phenotypic observation to a narrowed, mechanism-linked hypothesis.

Choice of model. Our aim was to establish single-cell 3D Cell Painting, and homogeneous spheroids provide a controlled, reproducible system in which to do so without confounding method development with cell-type heterogeneity. We did attempt organoids, but they do not fit our current automation: organoid loss is high enough that we can compute grit scores (Table1; Figure R1) but not reliably assess replicate behavior, and resolving this would require a new automation method — a project in its own right.

Cell-type vs cell-state heterogeneity. In our clonal spheroids there are no distinct cell types for identity markers to resolve, so we examined the heterogeneity that is present: using EdU, we found proliferation throughout the spheroid rather than confined to a rim (Fig. 6A). Such markers add the most value in genuinely heterogeneous models (organoids, co-cultures, non-transformed tissue), which we agree are compelling follow-up work.

Antibody integration (Cell Painting PLUS). The von Coburg et al. (2025) assay adds channels through a staining-elution step that strips and re-stains the same cells, requiring washing. Our workflow is incompatible, because the clearing needed to image deeper cells leaves the spheroid in a viscous fructose-glycerol medium that cannot be washed out. We report this, alongside organoid extension, as a priority future direction (Discussion, l. 627–28).

Table 1: Attrition of spheroids

compound	dose (µM)	type	planned	imaged	lost	kept %
**DMSO**	0.1	neg_con	38	14	24	37
**Berberine cl**	2.5	pos_con	10	4	6	40
**Etoposide**	2.5	pos_con	10	3	7	30
**Fenbendazole**	2.5	pos_con	10	3	7	30
**Abemaciclib**	1.0 / 3.0 / 10.0	trt	10 / 10 / 10	5 / 3 / 1	5 / 7 / 9	50 / 30 / 10
**Binimetinib**	1.0 / 3.0 / 10.0	trt	10 / 10 / 10	3 / 0 / 0	7 / 10 / 10	30 / 0 / 0
**Fluorouracil**	1.0 / 3.0 / 10.0	trt	10 / 10 / 10	1 / 5 / 2	9 / 5 / 8	10 / 50 / 20
**Gemcitabine**	0.1 / 0.3 / 1.0	trt	10 / 10 / 10	1 / 4 / 3	9 / 6 / 7	10 / 40 / 30
**Olaparib**	1.0 / 3.0 / 10.0	trt	10 / 10 / 10	7 / 3 / 4	3 / 7 / 6	70 / 30 / 40
**SN-38**	1.0 / 3.0 / 10.0	trt	10 / 10 / 10	4 / 5 / 2	6 / 5 / 8	40 / 50 / 20
**Trifluridine**	1.0 / 3.0 / 10.0	trt	10 / 10 / 10	7 / 5 / 4	3 / 5 / 6	70 / 50 / 40
**Veliparib**	1.0 / 3.0 / 10.0	trt	10 / 10 / 10	2 / 5 / 4	8 / 5 / 6	20 / 50 / 40

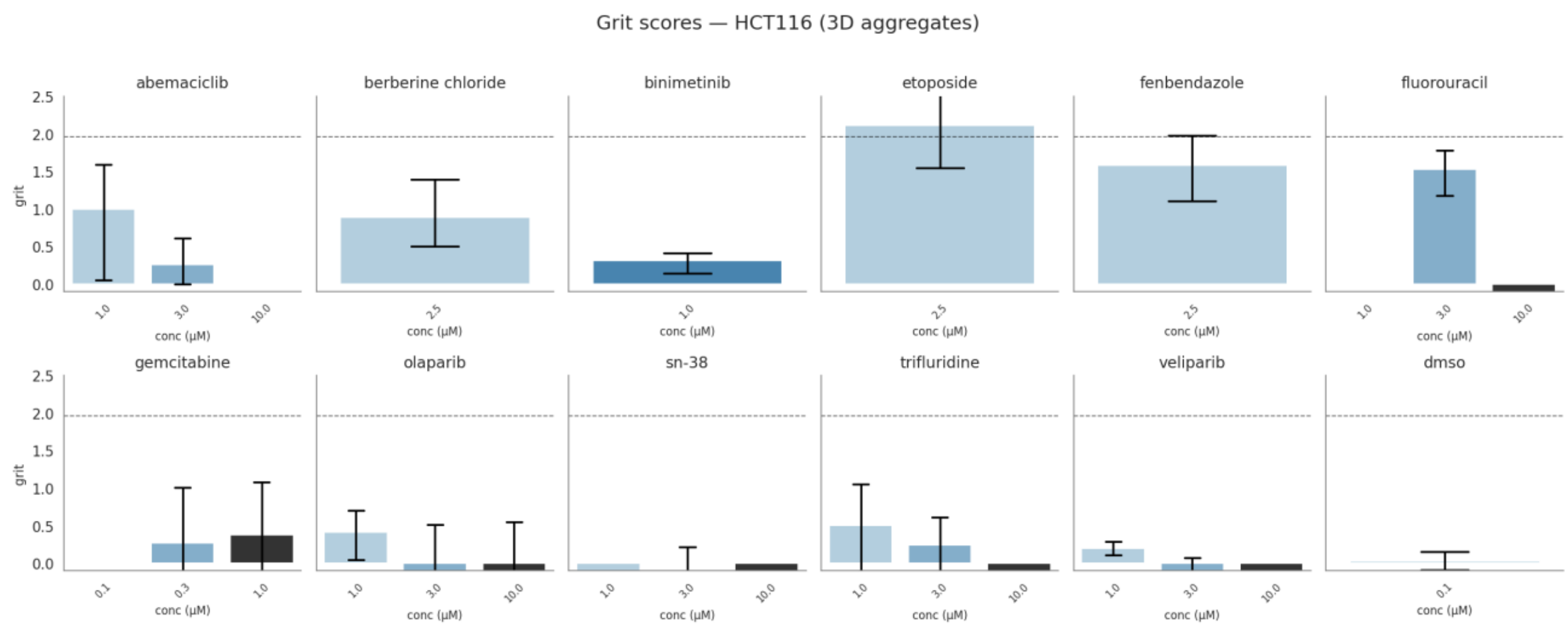

Reviewer Figure 1: Cell Painting of Apical-out organoid exposed to a selection of drugs.

2. Tissue clearing is brought as an essential part of the pipeline. However, since data is pooled one could scan more spheroids to reach the same conclusions. In particular since the imaging pipeline is fully automated, image acquisition could be easily expanded. Moreover, the authors even mention that water immersion already reaches higher mean FocusScore, questioning the need for the lengthy tissue clearing step. To evaluate the needs versus nice to haves, it is essential that investigations are performed into variables like impact of tissue clearing versus no clearing,

We agree it is important to separate genuine requirements from conveniences, and we have addressed the reviewer's clearing question directly. In our system, using ULA

plates, clearing is required to detect cells in the deeper layers of the spheroid: without it, detection falls off with depth (Supplementary Figure 3C).

On water immersion, the comparison reported in the manuscript was obtained on a different system, and a controlled comparison against clearing on matched hardware was not available to us. We therefore cannot resolve here whether it could substitute for clearing, and regard a clean comparison as worthwhile but separate work (Supplementary Methods, *Image acquisition*).

restricting image acquisition to lower ‘half’ of the spheroids/organoids (or predefined depths like 30um into a spheroid)

We investigated this. Acquisition is already restricted to the lower half of the spheroid, that is closer to the objective. Within this range, a single plane performs significantly worse than 6 sparse planes, while 9 and 12 planes perform no better (overlapping confidence intervals) (Supplementary Figure 3A1). Importantly, the deeper planes are retained; sparse spacing reduces sampling density without dropping depth. We interpret 6 sparse planes as an effective compromise, likely because at this spacing each cell is captured close to once rather than the three- to fourfold redundancy seen at finer spacing (Fig. 2E), while full depth coverage is preserved.

and compensating with scanning more objects, heterogeneity of cellular phenoscopes within and between independent spheroids/organoids (thus ideal number of cells analyzed per spheroid and number of spheroids) as well as the impact of relative positioning of cells within objects, and spheroid/organoid sizes (both in terms of biology as well as acquisition quality). Important is to define how do these variables combine for optimal data acquisition in terms of quality and quantity?

These variables are interlinked, and we agree that fully characterizing how they jointly optimize acquisition would be valuable. We tested several, though these comparisons (n=6) are powered only to detect large effects. Varying seeding density, and thereby spheroid size, reproducibility did not differ markedly, but the largest condition (810 cells/well) was weakest and showed qualitatively incomplete dye penetration (Supplementary Figure 3A3, A6). We therefore recommend a working range of approximately 100-150 µm, beyond which penetration is not guaranteed.

We have compiled all our findings, with the magnification, clearing, and plane-subsetting analyses, into concrete acquisition recommendations in the Discussion (l. 609). A full factorial optimization across the remaining variables, including the cells-per-spheroid versus spheroid-count tradeoff, would require a dedicated study, which we regard as important follow-up rather than within this manuscript's scope.

3. From a technical perspective, I understand that Cell Paint (cellpose + cell profiler) is applied on 2D excerpts (z-sections) that are taken from 3D spheroids. Can the authors generate a demonstrator dataset where the current pipeline (Cell paint on z-sections per 5um step size) is combined and compared with high resolution image analysis (40x) where cellular properties (cell profiler) are assigned to 3D rendered cell volumes (Nyquist step size acquisition)?

We thank the reviewer for this suggestion and have examined it from several angles.

We generated two additional datasets to probe resolution: spheroids imaged at 30× with finer axial sampling (1.5 µm spacing), addressing z-resolution, and images at 40×, addressing lateral resolution. Finer axial sampling gave negligible improvement over our sparse acquisition. The 40× data performed considerably worse despite the higher NA, likely because the thick round-bottom plates that make the method scalable also increase out-of-focus light, degrading signal-to-noise more at 40× than 30× (Supplementary Figure 3A5-A6). Neither finer axial sampling nor higher lateral magnification thus improved profiling in our system. This test does not, however, establish how true volumetric segmentation would compare, which we could not evaluate.

Full 3D volumetric segmentation also faces practical barriers on a spinning-disk confocal: the dense, near-isotropic z-stacks it requires introduce substantial photobleaching (Beghin et al., 2022) and inflate data volumes by roughly an order of magnitude, with little gain given that our coarser stacks perform equally well. A sparser pipeline is therefore far easier to implement, central to the accessibility our method aims for.

For precise spatial information, volumetric segmentation would likely be more accurate, but our method deliberately targets population-level profiling rather than spatial mapping. Where such 3D segmentation is the goal, light-sheet microscopy is the more appropriate platform, with excellent demonstrations in the literature, albeit at steeper setup cost and lower throughput. We have clarified this positioning in the Discussion (l. 591).

4. For a general reader, it will be informative to showcase how the dyes exactly stain individual cells in spheroids. High resolution images with 63x objectives would be informative.

The working distance of a 63X objective is not compatible with ULA well plates, and 40X images do not look representative of our system (Supplementary Figure 3). As an alternative, we preprocessed images by removing the background and revised the layout of Figure 2 per your suggestion to better visualize the individual stains.

Also, what is the impact of image resolution on the ultimate phenoscaping outcome?

On image resolution and its impact on phenotypic profiling, we tested three magnifications (n=6). At both 30× (20× objective with 1.5× physical zoom) and 10×, replicate profiles separated from the non-replicate null, indicating the assay resolved true replicate signal. At 40× they did not, with replicates failing to exceed their null, and we therefore do not recommend it (Supplementary Figure 3A5).

Minor comments:

Fig 2A-C don't add much to the main part of the manuscript, I would combine these in a single panel. Furthermore, adding high-resolution images of the individual dyes would add to clarity.

We rearranged the figures into a single panel, focusing more on the single cell morphology that we capture.

Fig 3A2 and B2: The bar graphs are hard to interpret, adding a threshold line for the grit score (> 1.96) can help with this.

We have inserted a threshold line, and indicated the threshold in the figure caption.

Fig 4A-B1: The UMAPs are nice, however the colors are hard to connect to the legends. Adding a number in combination with the colors would help.

We have implemented this helpful suggestion.

Fig 5 is nice to show the principle, however I believe it does not add much to the main rationale. Therefore, I would consider moving it to the supplement.

We have moved figure 5 to the supplementary figures and improved the method description.

Reviewer #4 (Remarks to the Author):

Comments for the Author

This manuscript extends Cell Painting to 3D spheroids by combining clearing, confocal optical sectioning, and automated single-cell analysis. The topic is timely and relevant for phenomics and preclinical screening. To further meet the journal's standards for rigor and reproducibility, several conclusions and figures would benefit from additional

methodological detail and quantitative support.

Major Comments

The study currently presents a qualitative segmentation example (e.g., Fig. 2E). To enhance robustness, consider adding a small expert-annotated set and reporting quantitative metrics across depths and channels (e.g., IoU/Dice/F1), together with a discussion of how segmentation error propagates to features and clustering.

We have added an expert-annotated set including the representative drugs Binimetinib, Abemaciclib, SN-38 and DMSO. The IoU across channels, depth and cell line is reported in Supplementary figure 3C-D. Notably the quantified overlap shrinks in deeper sections, and in Binimetinib. This drug affected the nuclear integrity, and is known to induce cell death in the spheroid core (Folkesson et al., 2020), a poor performance is not entirely unexpected. We then assessed how this error propagated to features and clustering by calculating the Pearson correlation between feature profiles of cellpose-segmented and expert-segmented objects. Feature-level Pearson correlations between Cellpose- and expert-derived profiles correlated strongly across all conditions (0.93-0.99), with the lowest value for Binimetinib nuclei (r = 0.93).

We included an explanation in the Methods, *expert annotations (l. 864).*

The wording "cells per spheroid" could be misinterpreted; it appears closer to a sum of per-section 2D segmentation instances, where the same cell may be counted across multiple sections. It may be clearer to phrase this as "2D segmentation object instances per spheroid," explicitly noting that this is not a unique 3D cell count; if feasible, a simple cross-section de-duplication estimate would be helpful.

We have adjusted the phrasing per your suggestion, and included a de-duplication estimate in Figure 2E. This estimate indicates a 3–4-fold duplication of each cell, bringing the number of cells per spheroid to ~400 (HCT116) and ~ 330 (HT29) cells per spheroid. We described the deduplication in the text per your suggestion (l. 200).

Per-section normalization assumes similar technical attenuation across wells at the same depth. When compounds markedly alter spheroid optics (e.g., strong necrosis or vacuolization affecting scattering/refractive index), this assumption may introduce bias. It would help to acknowledge this as a methodological limitation and include a few robustness checks.

We acknowledge that compound-induced morphological changes (Supplementary Figure 2C) affect optical properties, introducing potential normalization bias. As robustness checks, normalization removes the depth-dependent signal trend in controls (Figure 2G; Supplementary Figure 2A-B), peak focus depth stays reproducible across treatments and channels (Supplementary Figure 3C), and segmentation accuracy decreased but did not transfer to compound-treated feature profiles (Supplementary Figure 3D-E). We note this potential bias as a methodological limitation in the Discussion (l 623).

Fig. 2H. This figure is used to indicate a reduction of depth artefacts, but it is difficult to interpret rigorously in its current form. To make it more informative, please: (i) label axes with the percentage of variance explained by PC1/PC2 for both panels; (ii) clarify in the legend the granularity of each point (e.g., well-level aggregate vs per-section profile); and (iii) state whether the two panels use a shared PCA loading basis or independent PCAs (if independent, note that axes are not directly comparable). If appropriate, learning a shared basis on combined data and reporting PC–section correlations or a simple variance-partition analysis would strengthen the claim.

We thank the reviewer for the helpful quantitative suggestions. We have addressed all points as follows.

1) We have labeled axes with the percentage of explained variance.

2) The text and figure legend now explicitly indicate the aggregation level (per-section profiles)

3) We computed the PCA based on a shared loading to ensure that axes are directly comparable. This we have indicated in the text and figure scripts.

4) We have calculated correlations between principal components and section number, reporting these values in the text (PC2: $r = -0.79$, $p < 0.001$) and updated the figure.

In Fig. 4A1/B1, UMAP embeddings incorporate pathway labels at the embedding stage, which is helpful for visualization. To support statements about discovering pathway-level structure, it would be valuable to include fully unsupervised embeddings and quantify label enrichment (e.g., ARI/NMI, permutation tests). For reproducibility, please disclose key UMAP hyperparameters (n_neighbors, min_dist, metric, random_state) and provide the label file used for semi-supervision.

1) We have now included a fully unsupervised embedding in Figure 4A1-B1. The hyperparameters are n_components=2, spread=5, n_neighbours=200, min_dist=0.1, random_state=42, metric='cosine' as described in the methods.
2) The label file is now included in the supplementary files.

3) We quantified label enrichment (ARI, NMI) and performed permutation tests. These values are now included in the figure as well as the text.

For the comparison between Fig. 4A2 and 4B2, the characterization of the MIP heatmap as a "single cluster" feels somewhat subjective. It may be clearer to specify clustering rules (distance, linkage, tree-cut height or dynamic tree cut), report the resulting number of clusters and separation metrics (e.g., silhouette score), and—under the same sample filter (e.g., grit ≥ 1.96)—use pathway labels as external truth for both MIP and scAgg.

1) We have now included an objective characterization of clustering. Like the for the heatmaps, we computed a cosine similarity matrix (filtered by grit ≥ 1.96), applied the Ward linkage method, and extracted flat clusters based on a maximum average silhouette score (k=2-8). In addition, we quantified label enrichment (ARI) for those clusters.

k	MIP Silhouette	MIP ARI	scAgg Silhouette	scAgg ARI
**2**	**0.793**	**-0.013**	**0.457**	**0.041**
3	0.630	0.009	0.351	0.139
4	0.326	0.030	0.345	0.159
5	0.294	0.031	0.333	0.183
6	0.292	0.032	0.331	0.188
7	0.288	0.033	0.311	0.206
8	0.153	0.068	0.253	0.252

2) We observe the following: MIP clusters are cohesive but biologically arbitrary, while scAgg clusters are less cohesive but mechanistically more informative – increasing ARI from 0.041 (k=2) to 0.252 (k=8), indicating that spheroid aggregate profiles encode mechanism-of-action information better than MIPs.

In Fig. 6A, the text could be read as implying that lower grit in 3D directly reflects weaker biological responses. Since grit reflects both effect size and measurement noise, and 3D assays inherently face added noise (penetration, scattering, depth attenuation, segmentation uncertainty), it would strengthen the analysis to elaborate on these technical factors and, where possible, separate them from biology.

We thank the reviewer for the comment on the grit score reflecting both effect size and measurement noise. The manuscript should be clear about this.

We rewrote the text in line l. 440 to: "*Since the grit score reflects both effect size and measurement noise, lower grit scores could indicate both the inherent challenges of volumetric imaging as well as potentially weaker biological responses.*"

The differences shown in Fig. 6D–F are consistent with spatial proliferation and microenvironmental heterogeneity in spheroids. If the intent is to emphasize new mechanistic insight, some softening of language may help, alongside additional support such as depth-resolved proliferation readouts (e.g., EdU/Ki-67) or ring/zone morphology analyses to bridge morphology and mechanism.

We are grateful for this suggestion, which we shared at the outset. We expected a proliferating-rim and quiescent-core to contribute to the divergence (Figure 5D-F); to our surprise, EdU staining spanned the whole spheroid (Figure 6A), showing proliferation throughout rather than a spatial gradient (Freyer & Sutherland, 1985; Laurent et al., 2013). This result is what led us to hypothesize that the divergence instead reflects 5-FU and Olaparib engaging p53 through distinct mechanisms reshaped by the 3D format, as developed in the Discussion (l. 586). We thank the reviewer for helping us make this explicit.

To improve reproducibility, please provide and clearly link in the data availability/methods the following materials already referenced in the main text but not currently included with the submission: Supplementary Table 1 (compound list and doses) and Supplementary Table 2 (dyes/channels/filters/exposure settings).

We apologize for the inconvenience, the Supplementary tables are now added.

Minor Comments

Clarify inclusion/exclusion criteria across analyses (e.g., whether grit < 1.96 is uniformly excluded), so readers can compare figures consistently.

We have now implemented grit filtering (> 1.96) uniformly by updating the UMAPs. In the labeled UMAP (Figure 4A1-B1), datapoints with a grit lower than the threshold were made transparent. In the unsupervised UMAP, those datapoints were filtered out before embedding.

Maintain consistent terminology (e.g., choose between “spheroid-wide MIP” and “MIP-based profiles” and use it throughout).

We have adjusted the terminology to be consistent.

Improve figure legibility by adding scale bars, channel labels, and contrast adjustments, and indicate magnification and depth for all panels.

We improved figure legibility per your suggestions.

A few spelling/formatting items could be polished (e.g., “single-cel”).

We have polished the spelling/formatting items.

For code usability and reuse, please add a small reproducibility bundle: (i) an environment lock file (requirements.txt/environment.yml), (ii) a tiny public test dataset with a clear README (install, data paths, one end-to-end example), and (iii) a short script/notebook that regenerates a small subset of panels. If feasible, expose core steps as simple documented functions and include one or two sanity checks.

We have added a Code availability section.
The analysis notebooks, including an environment lock file, are at https://github.com/pharmbio/colopaint3D, and we have established a Code Ocean pod which serves exactly as a small reproducibility bundle. During the revision, the link should be available through the revision system. After publishing, the pod will receive a DOI and become public.

I hope these suggestions help strengthen the manuscript, and I look forward to seeing a revised version.

Reviewer #4 (Remarks on code availability):

At present, the repository functions more as analysis scripts than a turnkey reproduction, and I did not attempt a full end-to-end run. A brief README covering installation and data paths, an environment lock file (requirements.txt or environment.yml), and a very small public test dataset would make it easier to try the code end-to-end. If feasible, a short script or notebook that regenerates a small subset of panels from the released feature tables would further lower the barrier to reuse.

The repository and Code Ocean capsule are now cited in the Code availability section, providing what should be sufficient material to reproduce our analysis.

REVIEWERS' COMMENTS

Reviewer #1 (Remarks to the Author):

The revised manuscript has been extended to include further validations of the original findings. This reinforces my earlier recommendation for publication and I have no further comments.

We thank the reviewer for their kind words.

Reviewer #2 (Remarks to the Author):

I would like to congratulate the authors on the successful improvement of their manuscript. All raised issues have been satisfyingly addressed. The functional validation of the divergent profiles of the DNA-damage-response agents 5-Fluorouracil and Olaparib in 3D is impressive.
Publication in Nat Comm is warranted.

We thank the reviewer for their kind words.

Reviewer #3 (Remarks to the Author):

I thank the authors for their revisions and for the thorough and thoughtful responses to my comments and suggestions. I appreciate the explanation why the authors deliberately chose spheroids as their model system for developing an accessible and easily scalable high-throughput assay. At the same time, it is unfortunate that most of the suggestions to gain impact (like exploring applications on organoids, cell types etc) were textually argued to be beyond the scope of the paper. That may be true, but the current focus on spheroids significantly downplays the impact of the manuscript as the technical gains are more like refinements than a substantial novel methodology.
Second, a significant downfall of the manuscript is the absence of a direct comparison of their approach with a true volumetric 3D segmentation and downstream analysis strategy. While the authors clearly explain that volumetric imaging has significant disadvantages in terms of image acquisition and assay practicality, it now remains handwaving how much superior performance a true volumetric 3D analysis pipeline could achieve versus its costs in terms of big data, compute and practicality. A head-to-head comparison could have been

very instrumental in guiding future researchers to select their method of choice.
For instance, demonstrating that the presented method achieves (near)-equivalent outcomes to those obtained through true volumetric 3D segmentation, while being far superior in terms of practicality and scaling would substantially strengthen the impact of the paper and broader applicability and adoptability of their approach.

We thank the reviewer for this assessment. We agree that exploring applications on organoids, cell types etc. would lift the impact of the paper. As such, we attempted an organoid adaptation during revision but did not achieve the necessary organoid retention in the available time; we view this as a natural next step and have noted it as such, rather than a proof that the current spheroid-based approach lacks broader applicability, as we demonstrated that Cell Painting on spheroids can uncover different biology from Cell Painting on monolayers.
We agree that a direct comparison with true volumetric 3D segmentation would be informative and acknowledge this as a limitation of the current study. However, we note the trade-off is not unquantified: as detailed in the Discussion, matching our xy resolution in a fully resampled 3D volume would inflate data volumes ~20-fold to the tens-of-terabyte range per plate, which is impractical at the throughput scale (compound/dose panels across cell lines) this method targets. This trade-off — better spatially resolved, near-isotropic 3D segmentation at high cost and low throughput with possible bleaching, versus our less-detailed per-plane approach at practical scale — is precisely the choice we aim to make explicit.

Reviewer #4 (Remarks to the Author):

Thank you for the careful revision. Most of my previous concerns have been addressed. I only have a few remaining points.

We thank the reviewer for carefully checking our revised work. Here are our answers:

1. The depth-normalization analysis is clearer, but I am not sure whether the same DMSO controls were used both to calculate and evaluate the correction. If so, the complete loss of the depth trend would be partly expected. Please clarify this point and consider changing “remove” to “mitigate” if no independent validation was used.

We thank the reviewer for pointing out this circular reasoning. To address this concern, we have remade the figures after using a half of the DMSO wells for estimating the normalization mean, and half for visualizing and computing the before and after. We now have a real non-zero mitigation. “*Section-dependent effects are present before normalization*

(Figure 2g, left; r = 0.79, η^2 = 68 %) and mitigated after normalization (Figure 2g, right; r = 0.00, η^2 = 1%)". Note that the plot looks slightly different because fewer points have been used to make the plot.

2. Please clarify the hierarchical clustering method. The manuscript mentions Ward linkage together with cosine similarity, which is not a standard combination. A brief clarification, or a robustness check using a linkage method compatible with cosine distance, would be helpful.

We thank the reviewer for spotting this inconsistency. The Methods section described it wrongly. Pairwise compound similarity was computed as the cosine similarity between median consensus profiles (compounds with grit > 1.96). The resulting compound × compound similarity matrix was clustered by hierarchical clustering with Ward linkage on Euclidean distances between the rows of that matrix *(seaborn.clustermap, method='ward', default metric='euclidean')*, so that compounds are grouped by the similarity of their full similarity profiles. Now we updated the Methods to accurately convey this.

3. The new EdU, γH2AX, and RNA-seq data strengthen the biological interpretation. However, "p53-dependent" seems somewhat strong because HCT116 and HT29 are not isogenic. Please soften this wording.

We use HT29 as a TP53-mutant control following established previous work describing Nutlin (Tovar et al., 2006), and the loss of the MDM2-inhibitor grouping specifically in HT29 supports a p53-dependent interpretation. We agree HCT116 and HT29 are not isogenic and have already noted in the Discussion that this comparison differs in more than p53 status, and that attribution to p53 relies chiefly on the MDM2 inhibitor result. As such we have softened the wording to "segregate with p53".

4. The supplementary compound table is now included, but I could not find the exact four concentrations used for each compound in the main 2D and 3D screens. Please add these values or indicate clearly where they are listed.

We have indicated the concentrations in the Methods: 10μM, 3μM, 1μM, and 0.3μM for 3D and 10μM, 3μM, 1μM, and 0.3μM, 0.1μM for 2D.

The doses are also indicated per file as a metadata column in the image data repository (https://ftp.ebi.ac.uk/biostudies/fire/S-BIAD/254/S-BIAD2254/Files/spher_colo52-FileList.tsv)
