# Supplementary material for: High-content morphological profiling by Cell Painting in 3D spheroids
Source: Nat Commun. 2026 Oct 21;17:10790. doi: 10.1038/s41467-026-78596-5 (PMC13686983; doi:10.1038/s41467-026-78596-5)
Supplement: Supplementary file 5 [file 41467_2026_78596_MOESM4_ESM.docx]

**Supplementary Data 2.** Small molecule screening data

| **Category** | **Parameter** | **Description** |
| --- | --- | --- |
| Assay | Type of assay | A Cell Painting workflow for 3D spheroids. Multiplexed fluorescent staining of cellular compartments is imaged by spinning-disk confocal microscopy and quantified at single-cell resolution. |
|  | Target | No defined molecular target; target-agnostic profiling of whole-cell morphology in HCT116 and HT29 spheroids, with a 2D monolayer comparison. Methods, “Cell lines” and “2D Monolayer Cell Painting”. |
|  | Primary measurement | >2,000 morphological features per object across five fluorescence channels, aggregated to per-well profiles by two strategies: scAgg (object = cell) and MIP (object = spheroid; shape, intensity and texture only). Methods, “Feature extraction and preprocessing”; Supplementary Table 3. |
|  | Key reagents | Six-dye Cell Painting panel (Hoechst 33342, concanavalin A–AF488, SYTO 14, WGA–AF555, phalloidin–AF568, MitoTracker Deep Red FM): suppliers, catalogue numbers and concentrations in Supplementary Data 2. Fixation, permeabilization, clearing and culture reagents in Methods, “Cell lines”, “Clearing solution” and “The Cell Painting assay”; differences between arms in Supplementary Table 2. |
|  | Assay protocol | Methods, “3D Spheroid Cell Painting”, “3D Image acquisition”, “Spheroid detection method” and “Feature extraction and preprocessing”; overview in Figure 1. Adapted from Bray et al. (2016) and Cimini et al. (2023). Example dataset: BioImage Archive S-BIAD2254. |
|  | Additional comments | Method-development / proof-of-concept study rather than a hit-discovery campaign. Adaptations relative to standard 2D Cell Painting and second-site reproduction: Methods, “3D Spheroid Cell Painting”, “Spheroid detection method” and “Second-site validation”. |
| Library | Library size | 52 small molecules (Supplementary Data 1). |
|  | Library composition | Clinically annotated compounds curated for relevance to (colorectal) cancer therapy, spanning 14 pathway classes; per-compound annotation in Supplementary Data 1. Phenotypic reference compounds (etoposide, fenbendazole), cytotoxic control (staurosporine), negative control (D-sorbitol) and vehicle (DMSO) were included alongside the library. |
|  | Source | SelleckChem Cherry-Pick library; AMG-232 and 5Z-7-oxozeaenol from Sigma-Aldrich, as were the reference and control compounds. Per-compound supplier and catalogue number in Supplementary Data 1. |
|  | Additional comments | Stocks at 10 mM in DMSO (a small number in water), stored at −20 °C; four concentrations per compound. Methods, “Compound library and compound spotting”. |
| Screen | Format | 384-well ultra-low-attachment plates (Corning 3830), one spheroid per well; outer wells excluded, layouts designed with PLAID. 2D comparison on PhenoPlate 384-well plates (Revvity 6057302). HCT116 and HT29 with two biological and two technical replicates per condition; second site HT29 only, in triplicate (Supplementary Table 1). |
|  | Concentration(s) tested | 10, 3, 1 and 0.3 µM, with 0.1 µM additionally in the 2D arm; vehicle 0.1% DMSO. Second site: 10-point, 3-fold series from a 10 µM top concentration (Supplementary Table 1). |
|  | Plate controls | DMSO (0.1%) vehicle, D-sorbitol negative control, staurosporine cytotoxic control, and etoposide and fenbendazole as phenotypic reference compounds; 20 interspersed DMSO wells per plate. |
|  | Reagent/ compound dispensing system | I.DOT (compound spotting), ViaFlo 384 (seeding and compound transfer), BioTek 405 LS and MultiFlo FX (washes and reagent dispensing). Second site: Echo 650 and Multidrop (Supplementary Table 1). |
|  | Detection instrument and software | Nikon ECLIPSE Ti2-E spinning-disk confocal with NIS-Elements; Yokogawa CV8000 at the second site. Cellpose v2.0 cyto2 (retrained), CellProfiler 4.2.1 for scAgg and MIP or 4.2.5 for 2D, and Pycytominer (Supplementary Table 3). |
|  | Assay validation/QC | Focus-based spheroid detection (Methods, “Spheroid detection method”); PowerLogLogSlope image QC in the MIP pipeline; grit scores and median pairwise replicate correlations against a matched null (Methods, “Data analysis and visualization”). Cell lines mycoplasma-tested and STR-authenticated. |
|  | Correction factors | Per-optical-section standardization to matched-section negative controls in 3D; illumination correction in 2D. Methods, “Feature extraction and preprocessing”; Supplementary Table 3. |
|  | Normalization | As above, followed by clipping at ±40, feature selection and aggregation to per-well profiles (Supplementary Table 3). |
|  | Additional comments | scAgg versus MIP comparison: Results and Figure 3. |
| Post-HTS analysis | Hit criteria | No potency-based hit-calling. Phenotypic activity defined as grit > 1.96, the two-sided 95% critical value of the null distribution (Methods, “Data analysis and visualization”). |
|  | Hit rate | Of 52 compounds: HCT116 38 (MIP) / 37 (scAgg); HT29 33 (MIP) / 32 (scAgg); 2D monolayer 46 (HCT116) and 47 (HT29). Figures 3c–d and 5a. An activity benchmark, not a discovery hit rate. |
|  | Additional assay(s) | 2D monolayer Cell Painting, a targeted EdU / γH2AX assay in HCT116 spheroids, and bulk RNA-seq. Methods, “2D Monolayer Cell Painting”, “EdU and γH2AX assay” and “Bulk RNA-seq”. |
|  | Confirmation of hit purity and structure | Commercial vendor compounds used as received; all ≥98% pure per suppliers’ certificates of analysis (Methods, “Compound library and compound spotting”). No de novo confirmation of purity or structure. |
|  | Additional comments | See Discussion. |
